# HO-CR and HOLL-CR: new forms of winter oilseed rape (*Brassica napus* L.) with altered fatty acid composition and resistance to selected pathotypes of *Plasmodiophora brassicae* (clubroot)

**DOI:** 10.1007/s13353-024-00867-y

**Published:** 2024-04-19

**Authors:** Stanisław Spasibionek, Katarzyna Mikołajczyk, Marcin Matuszczak, Joanna Kaczmarek, Noor Ramzi, Małgorzata Jędryczka

**Affiliations:** 1 Plant Breeding and Acclimatization Institute-National Research Institute, Department of Oilseed Crops, Poznań, Poland; 2 Institute of Plant Genetics, Polish Academy of Sciences, Poznań, Poland

**Keywords:** *Brassica napus*, Fatty acid, HO lines, HOLL lines, *Plasmodiophora brassicae*, Clubroot, Disease resistance

## Abstract

The priority in oilseed rape (*Brassica napus* L*.*) research and breeding programs worldwide is to combine different features to develop cultivars tailored to specific applications of this crop. In this study, forms with a modified fatty acid composition of seed oil were successfully combined with a source of resistance to *Plasmodiophora brassicae* Wor., a harmful protist-causing clubroot. Three HO-type recombinants in F_6_–F_12_ generations with oleic acid content of 80.2–82.1% and one HOLL-type F_6_ inbred mutant recombinant (HOmut × LLmut), with a high oleic acid content (80.9%) and reduced linolenic acid content (2.3%), were crossed with the cultivar Tosca, resistant to several pathotypes of *P. brassicae*. The work involved genotyping with the use of DNA markers specific for allelic variants of desaturase genes responsible for the synthesis of oleic and linolenic fatty acids, CAPS (FAD2 desaturase, C18:1), and SNaPshot (FAD3 desaturase, C18:3), respectively. Of 350 progenies in the F_3_ generation, 192 (55%) were selected for further studies. Among them, 80 HO (≥ 72%) lines were identified, 10 of which showed resistance to at least one up to four *P. brassicae* pathotypes*.* Thirty lines in the selected progeny contained high oleic acid and less than 5% linolenic acid; eight of them belonged to the HOLL type conferring resistance to at least one pathotype. Two HO lines and two HOLL lines were resistant to four pathotypes. The resulting HO-CR and HOLL-CR inbred lines with altered seed oil fatty acid composition and resistance to *P. brassicae* represent unique oilseed rape material with the desired combination of valuable traits.

**Supplementary Information:**

The online version contains supplementary material available at 10.1007/s13353-024-00867-y.

## Introduction

The growing demand for a healthy oil source for human nutrition and biofuel production, as well as the utilization of rapeseed meal as high-quality livestock feed, has resulted in a significant increase in the global area under cultivation of oilseed rape, reaching 37.8 M ha in 2022. Its acreage surpassed 9 M ha in 2021/2022 in Canada, 8.3 M ha in India, 6.8 M ha in China, and 5.4 M ha in the European Union (FAOSTAT 2023). In Poland, a consistent increase in the cultivation area of winter oilseed rape has been observed in recent years. According to the Central Statistical Office, the sown area in 2023 exceeded 1 M ha.

Oilseed rape (*Brassica napus* L. var. *oleifera*, ssp. *napus*) has gained global acceptance primarily due to significant advancements in the quality of seed oil, characterized by the absence of erucic acid (C22:1), and seed meal with a very low glucosinolate content (Downey and Rakow 1987, Friedt and Luchs 1999, Leckband et al. 2002). The improved cultivars of oilseed rape are known as double-low, double-zero, or canola (McVetty and Duncan 2015). Elimination of erucic acid has considerably increased the content of monounsaturated oleic acid C18:1 (to about 62%) and the sum of polyunsaturated acids: linoleic acid C18:2 (to 20%) and linolenic acid C18:3 (to 10%). Only erucic acid-free winter oilseed rape cultivars grown worldwide provide oil with a well-balanced composition of unsaturated fatty acids, including oleic acid, linoleic acid, and linolenic acid. The 2:1 ratio of linoleic and linolenic acid in such oils contributes to beneficial nutritional properties (Wittkop et al. 2009; Wroniak and Rękas 2016). As a result of such improvement, rapeseed oil has become the third major vegetable oil worldwide, with an estimated annual production of 32.1 MMT (million metric tons) in 2022, after soybean (60.2 MMT) and palm oil (77.5 MMT) (FAOSTAT 2023). The global oil crop market is highly competitive, requiring constant improvements in oil quality to meet consumers’ demands. The objective of modifying seed oil quality is to enhance its nutritional and functional properties without further processing for specific end-use markets (Nath et al. 2016).

Considering its relatively high amount of polyunsaturated fatty acids, canola oil is less suitable for deep frying and industrial purposes. This is attributed to the potential formation of trans-fatty acid and oil oxidation during high-temperature treatments or long storage. Canola oil with high oleic acid content (HO, exceeding 72%) is valuable in human nutrition, as it reduces cholesterol and triglyceride levels, thereby improving blood cell viscosity and thus preventing embolism and cardiovascular diseases (Jones et al. 2014, Davis et al. 2022). At the same time, canola oil with high oleic and low linolenic (HOLL) acid contents is desirable due to its extended shelf life and stability at high temperatures (Matthäus 2006, Matthäus et al. 2011), making it an optimal raw material for biodiesel production (Pinzi et al. 2009). Linolenic acid oxidates easily, and it is unstable during frying. It has been shown that the reduced amount of linolenic acid in rapeseed oil extends its durability (Scarth and Tang 2006; Roszkowska et al. 2015); therefore, such oil is optimal for deep-fat frying important for fast-food applications (Barth 2009). In 2007, HOLL canola oil was defined by Maher et al. (2007) in new spring canola cultivars in Australia, as an oil containing more than 65% oleic acid and less than 3% linolenic acid. Subsequently, Canadian and Australian open-pollinated and hybrid spring HOLL cultivars, containing 68% oleic acid and 3% linolenic acid in seed oil, were released by Cargill and described by Salisbury et al. (2009). The current industry standard definition of HOLL (or HOLLi) canola includes cultivars producing oil with more than 75% oleic acid and approx. 3% linolenic acid.

In Poland, chemical mutagenesis using ethyl methanesulfonate was applied to obtain mutant lines of winter rape that produce oil with a high content of oleic acid (C18:1, over 75%) and a low content of linolenic acid (C18:3, approx. 3%) (Spasibionek 2006). The initial HO and LL mutant lines showed low agricultural value (Spasibionek 2006), which was not only due to the toxic effects of chemical mutagenesis resulting from EMS treatment, but also due to successive inbreeding of canola over generations (Singer et al. 2016). However, persistent and dedicated research and breeding initiatives have yielded several new recombinant lines with altered fatty acid composition in the oil and improved seed yields in field trials. An example of an HO cultivar with over 79% oleic acid content in seed oil was cv. Polka (Spasibionek 2013), approved by the Research Centre for Cultivar Testing (COBORU) in 2018 and included in the National List of Agricultural Plant Varieties (http://www.coboru.pl). The procedure for selecting new recombinants with changed fatty acid composition in seed oil based on phenotypic analysis proved inefficient due to its high dependence on variable environmental conditions (Spasibionek 2004, 2013). Molecular markers, including CAPS (FAD2 desaturase, C18:1) (Matuszczak et al. 2020; Spasibionek et al. 2020) and SNaPshot (FAD3 desaturase, C18:3) (Mikołajczyk et al., 2010; Spasibionek et al. 2020), were successfully used to identify mutated alleles of desaturase genes responsible for the synthesis of oleic and linolenic fatty acids, respectively.

Clubroot is caused by the obligate protist *Plasmodiophora brassicae* Wor., one of the most destructive soil-borne pathogens which attacks over 3700 species (Dixon 2009). Severe disease symptoms manifest on numerous plants of the family Brassicaceae, including economically important spring (canola) and winter oilseed rape cultivars (Dixon 2014). The disease has been ranked among the top 10 most significant global threats to oilseed rape production (Zheng et al. 2020). In fields heavily infested with *P. brassicae*, yield reductions ranging from 30% to total crop loss have been reported (Strelkov et al. 2007; Hwang et al. 2010). Intensive oilseed rape production over the decades has caused substantial yield losses in Canada, China, and Europe, often attributed to clubroot (Donald et al. 2006; Chai et al. 2014; Diederichsen et al. 2014; Donald and Porter 2014; Peng et al. 2014). In Poland, the disease has been reported over a large area of oilseed rape cultivation (Korbas et al. 2009; Řičařová et al. 2016), but its severity depends on field history and location (Robak and Gidelska 2009; Jedryczka et al. 2014; Czubatka-Bieńkowska et al. 2020). Studies conducted between 2013 and 2019 have revealed that *P. brassicae* is present in agricultural soils across all 16 provinces of Poland (Czubatka-Bieńkowska et al. 2020). Recently, the disease has also been reported in South America (Botero et al. 2019; Padrón-Rodríguez et al. 2022). A worldwide map of clubroot incidence, created by Javed et al. (2023) based on recent scientific publications, demonstrated its presence on all continents, including Australia and Africa.

The global outbreak of clubroot is a consequence of the very intensive cultivation of oilseed rape associated with the frequent use of this crop in plant rotations (Robak 1994; Dixon 2009; Zamani-Noor et al. 2019). Many reports have demonstrated a strong correlation between clubroot incidence and low soil pH (Hamilton and Crête 1978; Gossen et al. 2014). Plants growing in highly alkaline soils exhibited the least damage caused by clubroot (Dixon 1991). The seedling stage was found to be the crucial period of particular susceptibility of plants to the pathogen (Struck et al. 2022). On the other hand, studies conducted in the Czech Republic, Germany, Poland, and Sweden indicated a low to moderate correlation with soil pH (Zamani-Noor et al. 2022). Additionally, some outbreaks of clubroot on agricultural soils with pH close to neutral have already been reported (Řičařová et al. 2016). Nation-wide maps of clubroot incidence have been developed in Germany and Poland (Zamani-Noor 2017, Czubatka-Bieńkowska et al. 2020), while in Canada, similar maps have been elaborated for provinces where the disease was most intense (Government of Alberta 2022; Government of Saskatchewan 2022) or newly emerging (Government of Manitoba 2022). Comparative experiments have demonstrated that the history of oilseed rape cropping and geographic origin affect the genetic structure of many *P. brassicae* populations. The pathogen itself exhibits genetic diversity (Strehlow et al. 2014), and its distribution may be area-specific (Strelkov et al. 2016; Pang et al. 2020).

Various methods have been employed to reduce the severity of clubroot disease, but none of them have proven sufficient to eradicate the pathogen. Clubroot management has always been a great challenge, and many agronomic and chemical measures have already been tested (Struck et al. 2022). While biofungicides were initially considered promising, biocontrol agents were generally found to be insufficient under high disease pressure (Narisawa et al. 2005; Peng et al. 2011a, b). Given the considerable differences observed between communities of microorganisms of healthy and clubroot-diseased plants (Lebreton et al. 2019), research focused on microbial composition. Certain carbon and nitrate sources have been identified as factors that reshape the initial microbial community, triggering the germination of *P. brassicae* resting spores and initiating the infection. Wang et al. (2023) have suggested that these are the soil bacterial communities rather than root exudates that regulate the germination of *P. brassicae* resting spores. The study revealed significant differences in the composition and abundance of bacterial taxa between resting spore-stimulating communities and non-stimulating ones. This discovery holds promise for developing novel strategies of clubroot disease control based on the deliberate breaking of *P. brassicae* spore dormancy in soil. The functional similarity of soils with reduced resistance across many agroecosystems suggests the possibility of developing soil microbiomes that suppress diseases (Raaijmakers and Mazzola 2016). Microbiome engineering seems to be a possible approach in the future (Mueller and Sachs 2015), and fertilizers enriched with beneficial bacterial strains (biofertilizers) have recently been proposed as a viable solution in agricultural practice (Mącik et al. 2020).

Breeding for resistance is the most desirable, economical, and environmentally friendly strategy of clubroot management (Rahman et al. 2014; Zamani-Noor et al. 2022). According to Wallenhammar et al. (2021), the recommendation to use clubroot-resistant cultivars of oilseed rape in integrated pest management programs should be based on the abundance of *P. brassicae* DNA in soils, with a threshold of 1300 gene copies per 1 g of soil. Clubroot resistance in oil seed rape cultivars still used commercially to this day is derived from the cultivar Mendel (Diederichsen et al. 2009) and is located on chromosome A03. The cultivar served as the first-generation resistance in oilseed rape until it was broken in the regions of intensive oilseed rape production, primarily in Europe and Canada (Zamani-Noor 2017, Strelkov et al. 2018, Zamani-Noor et al. 2022). A mapping study performed by Fredua-Agyeman and Rahman (2016) suggested the control of clubroot by a single dominant gene. This type of disease control is subject to cycles of growth and decline and can easily be lost under strong disease pressure (Mundt 2014). Copy number variation or presence-absence variation is often a natural mechanism for gaining or losing disease resistance (Gabur et al. 2020). In clubroot, local duplication of the resistance gene at the *Crr3* locus greatly contributed to the resistance of the oilseed rape cultivar Tosca, which also served as a good resistance source located on chromosome A03 (Kopeć et al. 2021). Subsequently, other genes harboring resistance to novel pathotypes were localized on other chromosomes (Hasan and Rahman 2016; Karim et al. 2020; Tonu et al. 2023). Their efficacy varied depending on the pathotype, and cautious breeding was needed to avoid loss of resistance during the production of doubled haploid lines (Fredua-Agyeman et al. 2018). Marker-assisted selection has resulted in the development of novel clubroot-resistant lines of oilseed rape effective against Japanese field isolates (Kawasaki et al. 2021). New resistance sources have also been found in Chinese cabbage (Niemann et al. 2017) and radish (Gan et al. 2022). Moreover, quantitative resistance found in *B. oleracea* has been increasingly studied (Wagner et al. 2019) and used as a source of clubroot resistance (Peng et al. 2018; Ce et al. 2021).

The aim of this study was to achieve a twofold improvement of oilseed rape, by combining high oleic (HO) lines with resistance to *P. brassicae* (clubroot) and a threefold improvement by combining high oleic and low linolenic (HOLL) lines with resistance to clubroot. Each time, the resistance was studied using six isolates of *P. brassicae.* To our knowledge, such a highly desirable combination of valuable traits has not yet been reported in oilseed rape thus far.

## Materials and methods

### Plant material

The development of HO and HOLL recombinants involved crossing high-yielding cultivars with mutant lines with high oleic acid content (HOmut ≥ 76%) and low linolenic acid content (LLmut ≤ 3%) previously obtained by Spasibionek (2006). The plant material used in this study included the clubroot-resistant (CR) winter oilseed rape cultivar Tosca, showing resistance to *P. brassicae* (Frauen 1999), three high oleic (HO type, 3038, 2050, and 2065) recombinant inbred lines from the F_6_–F_12_ generations, with a very high oleic acid content (up to 82.1%), and recombinant F_6_ inbred HOLL (HOmut × LLmut) mutants (2103), with high oleic acid content (80.9%) and low linolenic acid content (reduced to 2.3%). The breeding lines with changed fatty acid composition served as the maternal plants in crosses with the cultivar Tosca as the paternal form; the cultivar was originally bred by Svalöf Weibull, Sweden. Characteristics of the four mother genotypes used for crossings with the cultivar Tosca, including fatty acid content and allelic variants of FAD2 and FAD3 desaturases in the A and C genomes of *B. napus*, are presented in Table 1. The progeny of the crosses is referred to as “lines,” while the groups of lines differing with fatty acids or resistance and susceptibility are referred to as “forms.”

**Table 1:** Oilseed rape forms used for crossings in this study

Genotype	Origin	Fatty acid type	Generation	Fatty acid content (%)		Marker-assisted selection	
				Oleic C18:1	Linolenic C18:3	FAD2_A/C haplotype	FAD3_A/C haplotype
2038	HOmut (M10464) × Contact	HO	F12	80.2	6.6	Mut/wild	Wild/wild
2050	HOmut (M10453) × Contact	HO	F10	80.3	6.4	Mut/wild	Wild/wild
2065	Californium × HOmut (M10464)	HO	F7	82.1	5.6	Mut/wild	Wild/wild
2103	(LLmut (M681) × HOmut (M10464)	HOLL	F6	80.9	2.3	Mut/wild	Mut/mut
Tosca	SW seed, SE	canola	cultivar	62.0	10.0	Wild/wild	Wild/wild

*HO* high oleic form of oilseed rape, *HOLL* high oleic and low linolenic form of oilseed rape, *Tosca* double improved winter oilseed rape cultivar, *LLmut* low linolenic acid mutant genotype, *HOmut* high oleic acid mutant genotype, *Mut* mutated alleles, *Wild* wild-type, non-mutated alleles

Winter canola plants were vernalized for 8 weeks at 4 °C to induce flowering, and the following crosses were made: 2038 (HO type) × Tosca, 2050 (HO type) × Tosca, 2065 (HO type) × Tosca, and 2103 (HOLL type) × Tosca, as indicated in Table 1. The F1 plants resulting from the crosses with cv. Tosca were grown in the greenhouse until maturity, and their seeds were harvested. Populations of the F_2_ and F_3_ generations were grown in the field and evaluated for growth habit, earliness of flowering, and seed quality traits, including the content of seed oil, glucosinolates, and seed oil fatty acid composition. In each generation, self-pollination of individual plants was carried out by bag isolation to obtain the population of the next generation. Of the 350 recombinants obtained, 192 F_3_ breeding lines, as well as resistant and susceptible controls (cv. Tosca and cv. Polka, respectively), were evaluated for their resistance to six isolates of *P. brassicae* belonging to six pathotypes.

### Marker-assisted selection

Genomic DNA was isolated from young leaves using a modified CTAB method (Doyle and Doyle 1990), as described by Mikołajczyk et al. (2012). Quality and quantity of DNA samples were assessed on 0.8% agarose gel using 50 ng of undigested lambda DNA as a reference or by A260 and A280 UV absorbance measurements (Mikołajczyk et al. 2012). Allele-specific CAPS markers (Falentin et al. 2007) were used to monitor mutant and wild-type alleles of the functional desaturase gene BnA.FAD2, involved in oleic acid synthesis, in the A genome of *B. napus* (Østergaard and King 2008), as described by Matuszczak et al. (2020). PCR amplification products were analyzed using 1.4% agarose gel electrophoresis and scored as FAD2_A for wild-type and fad2_a for mutant alleles of the desaturase gene BnA.FAD2. Wild-type and mutant alleles of FAD3 desaturase genes in the A and C genomes of *B. napus* (BnA.FAD3 and BnC.FAD3, respectively) were scored as FAD3_A and FAD3_C for wild-type alleles and fad3_a and fad3_c for mutants using an allele-specific SNaPshot assay involving two steps: PCR amplification of short regions containing possible mutation sites, followed by microsequencing (Mikołajczyk et al. 2012) (Table 1).

### Biochemical analysis

#### Determination of fatty acids

The composition of fatty acids was determined by gas chromatography using a Hewlett Packard chromatograph, Agilent Technologies 6890N Network GC System. Fatty acids were extracted from the seeds using hexane, and subsequently, methyl esters of the extracted fatty acids were obtained. Separation of esters was performed using a DB-23 capillary column with a length of 30 m. Hydrogen was used as a carrier gas, with a column temperature of 200 °C and a detector temperature of 220 °C. The separation time was approximately 10 min. The course of chromatographic separation was recorded, and the percentage of individual fatty acids was calculated using Chemstation software.

#### Determination of glucosinolates

The content and composition of glucosinolates were determined by gas chromatography. Glucosinolates were extracted from seeds using methanol with barium acetate. Subsequently, silyl derivatives of desulfoglucosinolates were obtained, and total glucosinolate content (expressed in μmol/g of seeds) was analyzed. In this method, the European standard CRM-366, with a total glucosinolate content of 12.1 µM g^−1^ seeds and a tolerance of 0.8 µM g^−1^ seeds, was used to calibrate the chromatograph. This standard was developed by the Community Bureau of Reference (BCR) as an average value of ring-test analyses between eighteen laboratories.

### Preparation of single-club isolates of *Plasmodiophora brassicae*

Root samples showing clubroot symptoms were collected from commercial oilseed rape fields, with their origin detailed in Table 2. The collection and maintenance of the clubs were conducted following the procedures outlined by Řičařová et al. (2016). Resting spores were extracted based on the method proposed by Tewari et al. (2005), with additional washing with distilled water as described by Strelkov et al. (2006) and further modifications implicated in this study. Briefly, small parts of the clubs were blended in distilled water, and the resulting suspension was filtered through six layers of cheesecloth. Resting spore extractions were conducted at room temperature (20 °C). The spore suspension was examined using a hemocytometer (Bright-Line™, Merck Z359629), and spore concentration was adjusted to a final concentration of 1 × 10^7^ spores/ml, which was used for the inoculation of the susceptible *B. rapa* cv. Granaat. Each of the collected clubs was treated as a separate isolate. One randomly selected club was further fragmented and used for the inoculation using the above-described method. Three rounds of inoculation were performed, with each round randomly selecting a single club for further propagation in a sterilized soil substrate. The fourth round of inoculation was used to propagate the isolate again using *B. rapa* cv. Granaat, which was then regarded as a single-club isolate, similar to a genetically identical single-spore isolate.

**Table 2:** Characterization of *Plasmodiophora brassicae* isolates used in this study

Isolate no	Locality	Province	Geographical coordinates	Year
1	Krymławki	Warmia-Masuria	N 54° 13′ 14.7″; 21° 16′ 08.5″	2017
2	Siestrzechowice	Opole	N 50° 25′ 45.3″; 17° 15′ 48.8″	2019
3	Skołoszów	Subcarpathia	N 49° 56′ 39.1″; E 22° 48′ 24.4″	2018
4	Słoszewy	Kuyavia-Pomerania	N 53° 12′ 30.7″; E 19° 14′ 57.0″	2016
5	Olszyny	Podlasie	N 53° 12′ 45.1″; 22° 14′ 56.5″	2019
6	Wierzbiny	Świętokrzyskie	N 50° 42′ 09.4″; E 21° 39′ 15.5″	2018

#### Determination of *Plasmodiophora brassicae* pathotypes

Pathotype classification of *P. brassicae* populations was conducted on a set of 16 *Brassica* hosts (genotypes), including the European Clubroot Differential (ECD) set (Buczacki et al. 1975; Somé et al. 1996). Each population was inoculated separately onto the clubroot-resistant oilseed rape cv. Mendel, as proposed by Zamani-Noor et al. (2022). Seeds of the differentials were sown in multipots DP7/28 (PPH Roko, Piotrów, Poland), with dimensions of 66 × 66 × 70 mm. Each pot contained 5 seeds, and there were 4 pots per replicate (20 plants), with two replicates (2 × 20 plants). Small seedlings of 5-week-old plantlets were inoculated with a spore suspension at a concentration of 1 × 10^7^ spores/ml. The soil substrate (Klassmann-Deilmann, Geeste, Germany) was mixed with peat pH 5.5 (Biovita Ltd., Słomniki, Poland) at a ratio of 2:1. Plants were grown in a greenhouse for 5 weeks at 20 °C ± 2 °C. In the first week after inoculation, the soil was saturated with water and then fertilized with Florovit (Agrosimex, Goliany, Poland) and watered as needed. Twenty seedlings of each host line were inoculated with each population of *P. brassicae.* Inoculation was done using six isolates collected in 2016–2019 in six provinces of Poland (Table 2).

### Evaluation of clubroot resistance

Six weeks after inoculation, the plants were removed from their pots, roots were washed and evaluated for clubroot severity on a 0–4 scale (Kuginuki et al. 1999), where 0—no galling, 1—a few very small galls, 2—small galling on the main and lateral roots, 3—moderate galling, and 4—root totally deformed, turned into galls, only residual remains of the root visible. The Disease Severity Index (DSI) was calculated for each experimental variant using the formula of Horiuchi and Hori (1980), modified by Strelkov et al. (2006). The mean DSI of each host over twenty replicates (plants) was calculated for each *P. brassicae* population. DSI data were converted to susceptible or resistant to describe the resistance and susceptibility patterns for each plant genotype. A differential line was considered resistant when DSI was less than or equal to 25%, and it was considered susceptible when DSI was greater than 25%, as proposed by Somé et al. (1996). Statistical analysis was carried out using Statistica v. 7.0 (StatSoft Inc., USA) and Microsoft Excel 2010 (Microsoft, Inc., USA).

## Results

### Altered fatty acids

Crosses of high oleic (HO) or high oleic and low linolenic (HOLL) genotypes with the cv. Tosca, subsequently propagated to the F_2_ generation, yielded 350 recombinants, 192 of which (55%) were randomly selected for further propagation. The progenies of the selected 192 recombinant plants of the F_3_ generation were assessed for fatty acid composition, as well as the resistance to six isolates of *P. brassicae.* On average, the amount of oleic acid (C18:1) in the selected lines was 71.54%, and the amount of linolenic acid (C18:3) was 7.73%. The lines differed significantly in both oleic acid (62.2–82.6%) and linolenic acid (2.8–10.4%) contents in seed oil (Table S1).

**Fig. 1:**
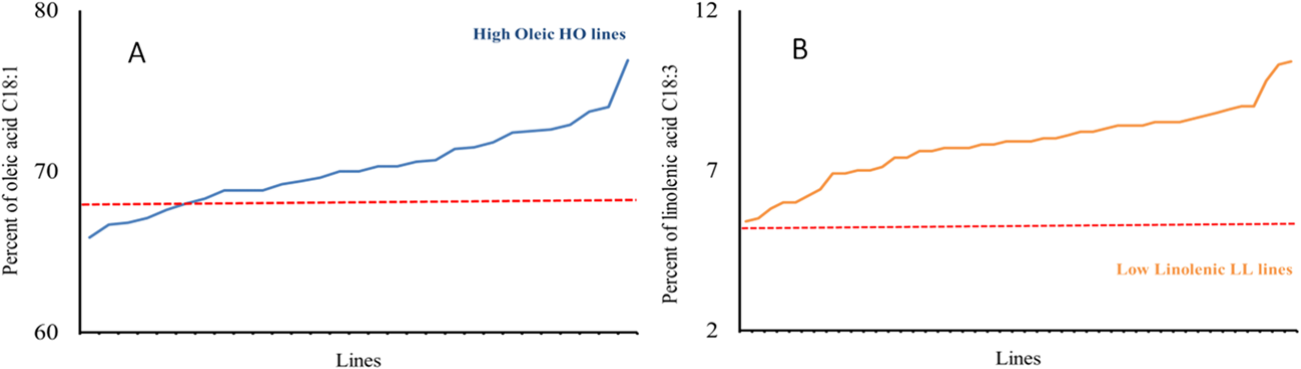
Content of oleic acid, C18:1 (**A**), and linolenic acid, C18:3 (**B**), in the F3 progeny of the genotype 2038 [HOmut (M10464) × Contact] crossed with the cultivar Tosca

**Fig. 2:**
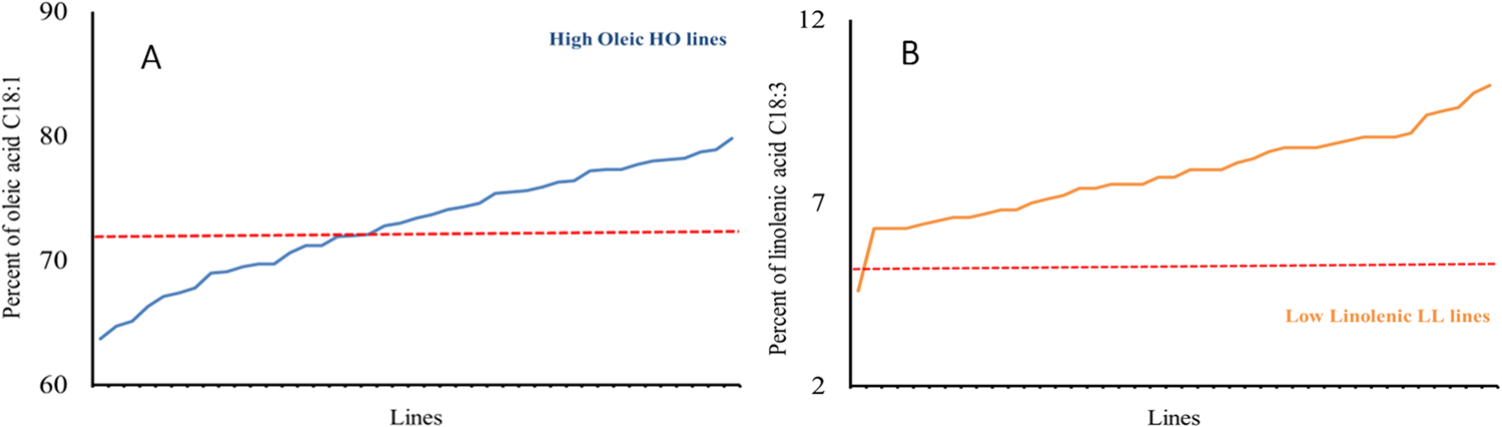
Content of oleic acid, C18:1 (**A**), and linolenic acid, C18:3 (**B**), in the F3 progeny of the genotype 2050 [HOmut (M10453) × Contact] crossed with the canola-type cultivar Tosca

**Fig. 3:**
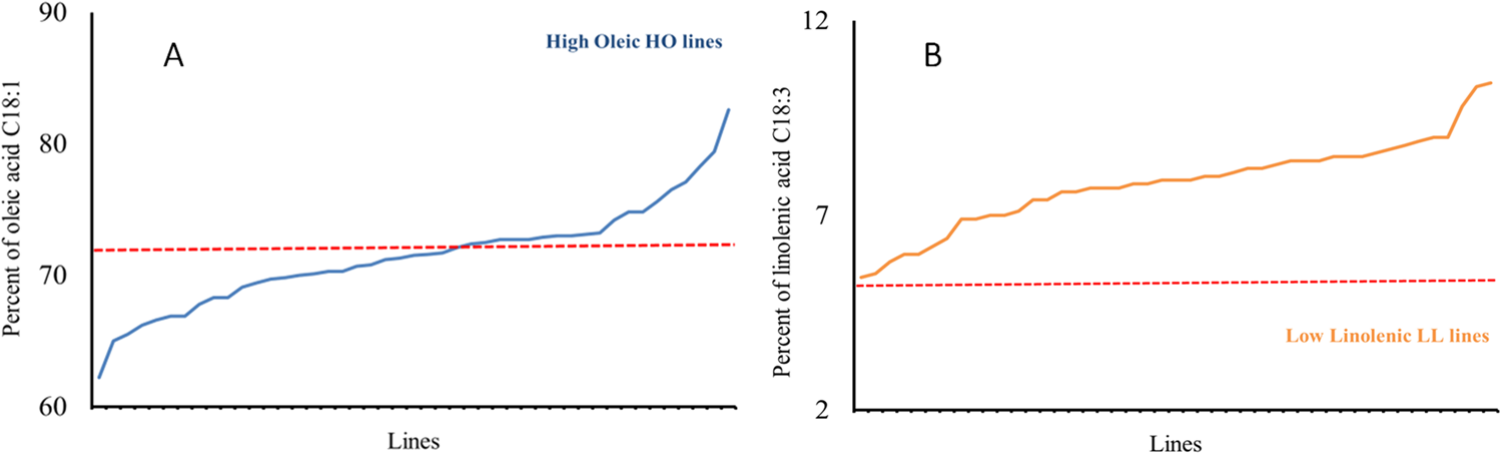
Content of oleic acid, C18:1 (**A**), and linolenic acid, C18:3 (**B**), in the F3 progeny of the genotype 2065 [Californium × HOmut (M10464)] crossed with the canola-type cultivar Tosca

**Fig. 4:**
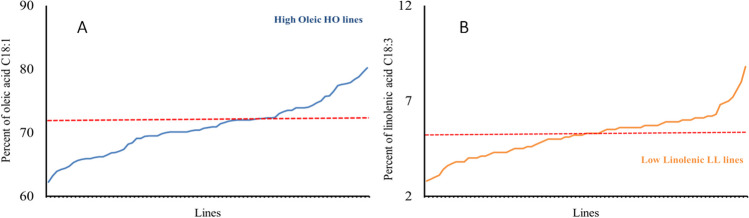
Content of oleic acid, C18:1 (**A**), and linolenic acid, C18:3 (**B**), in the F3 progeny of the genotype 2103 [LLmut (M681) × HOmut (M10464)] crossed with the canola-type cultivar Tosca

The canola-type cultivar Tosca showed the lowest content of oleic acid (62.0%) and one of the highest linolenic acid contents (10.0%). From the population of 192 lines, a group of 80 HO lines (72.1–82.6%) with an oleic acid content above 72% (Figs. 1A, 2A, 3A, and 4A) and a group of 30 LL lines (2.8–4.9%) containing less than 5% linolenic acid in the seeds were identified (Figs. 1B, 2B, 3B, and 4B).

**Fig. 5:**
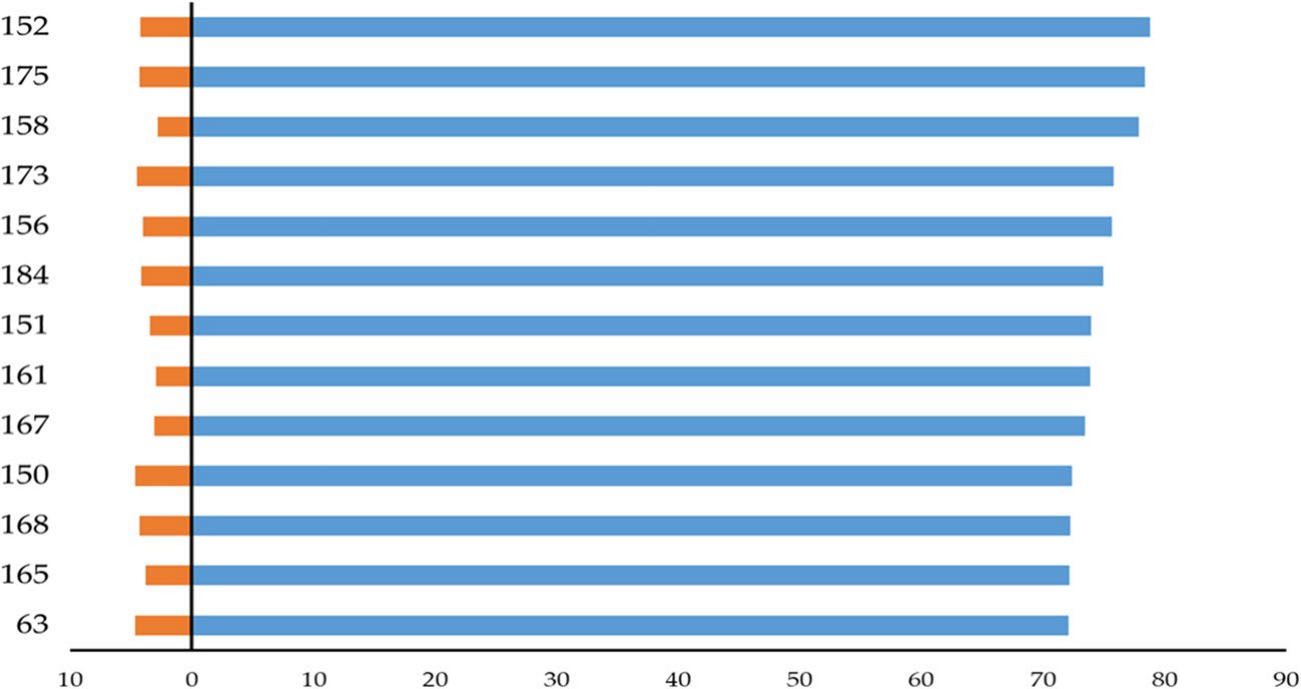
Content of oleic acid, C18:1 (blue), and linolenic acid, C18:3 (orange), in oilseed rape HOLL lines with high oleic acid and low linolenic acid

The most desired forms with altered fatty acids are the HOLL-type lines with a high content of oleic acid (≥ 72%) and less than 5% of linolenic acid in seed oil. Of the 192 lines tested, including 77 lines originating from the cross containing low linolenic mutant genotype as the component, the desired population of 13 lines met that condition. The content of oleic acid ranged between 72.1 and 78.8%, and the reduced content of linolenic acid ranged between 2.8 and 4.6% (Fig. 5). Twelve lines originated from the [LLmut (M681) × HOmut (M10464)] cross, which is the expected result. Interestingly, one line (no. 63) originating from the [HOmut (M10453) × Contact] cross contained 72.1% of oleic acid and 4.6% of linolenic acid, thus meeting the requirements of the HOLL type. Nevertheless, both values were very close to the threshold (Fig. 5).

### Determination of *Plasmodiophora brassicae* pathotypes

Pathotypes of *P. brassicae* were determined using two identification systems. According to Somé et al. (1996), the three pathotypes P1 (isolates 2 and 5), P2 (isolate 3), and P3 (isolates 1, 3, and 4) were identified. Using the ECD set** (**Buczacki et al. 1975), the following pathotypes were identified: 16/02/29 (isolates 1 and 3), 16/19/08 (isolate 2), 16/14/12 (isolate 4), 16/03/08 (isolate 5), and 16/15/15 (isolate 6). Of six isolates tested, five distinct pathotypes were designated: isolates 1 and 3 represented pathotypes P3 by Somé and 16/02/29 by Buczacki; isolate 2 belonged to P1 and 16/19/08; isolate 4 belonged to P3 and 16/14/12; isolate 5 belonged to P1 and 16/03/08; isolate 6 belonged to P2 and 16/15/15. The isolates did not infect the clubroot-resistant oilseed rape cv. Mendel.

### Clubroot resistance

**Fig. 6:**
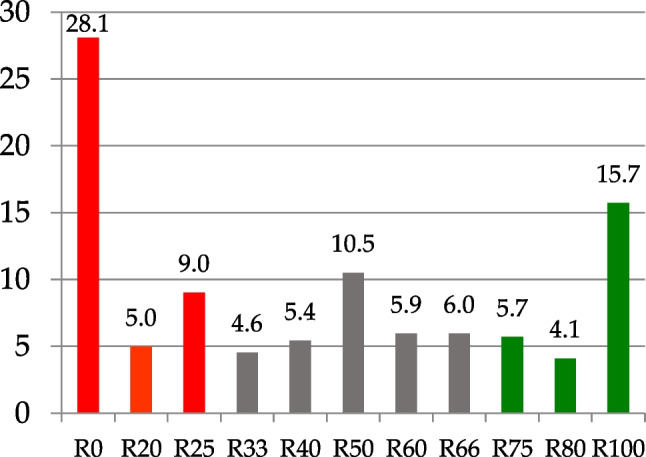
The average percentage of oilseed rape (*Brassica napus*) lines segregating for resistance to clubroot. The analyzed 192 lines resulted from the crosses between clubroot susceptible maternal lines, with high oleic (HO) and low linolenic (LL) acid content in seed oil and the canola-type clubroot-resistant cv. Tosca. The lines segregated with respect to resistance against *Plasmodiophora brassicae* (clubroot), ranging from R0 (fully susceptible) to R100 (fully resistant). The R20, R25, R33, R40, R50, R60, R66, R75, and R80 categories indicate lines with 20%, 33%, 40%, 50%, 60%, 66%, 75%, and 80% of plants resistant to the given pathotype of *P. brassicae*. R0, R20, and R25 are marked in red (fully or highly susceptible); R100, R80, and R75 are marked in green (fully or highly resistant)

In phytopathological tests conducted under greenhouse conditions, the clubroot-resistant cv. Tosca was fully resistant to pathotypes 4–6 and partially resistant to pathotypes 1–3 (67–75% of resistant plants), while the HO and HOLL forms used for crossbreeding were susceptible to all pathotypes. The selected 192 lines differed significantly in their resistance to the pathotypes tested, with ratios of resistant (R) to susceptible (S) plants varying between two extremes: from all resistant to all susceptible (Tab. S1-S4). There were 15 lines fully resistant to pathotype 1 and 7, 17, 55, 45, and 40 lines resistant to pathotypes 2–6, respectively (Table 3), i.e., an average of 30 R lines. The corresponding number of lines fully susceptible to pathotypes 1–6 was 55, 107, 40, 30, 34, and 55, averaging 53.5, which was 1.8 times the number of fully susceptible lines compared to fully resistant lines (Fig. 6).

**Table 3:** Proportion of lines resistant (R), susceptible (S), and segregating (R:S) to clubroot (*Plasmodiophora brassicae*) in the studied population of 192 oilseed rape (*Brassica napus*) recombinant lines resulting from the crosses of maternal plants—lines with HO or LL components and a paternal plant, the canola-type clubroot-resistant cv. Tosca

Ratio R:S	Isolate 1		Isolate 2		Isolate 3		Isolate 4		Isolate 5		Isolate 6	
	Pathotype P3; 16/02/29		Pathotype P1; 16/19/08		Pathotype P3; 16/02/29		Pathotype P3; 16/14/12		Pathotype P1; 16/03/08		Pathotype P2; 16/15/15	
	No	%	No	%	No	%	No	%	No	%	No	%
0:100	55	28.95	107	55.73	40	20.84	30	15.72	34	17.89	55	29.41
20:80	8	4.21	18	9.38	15	7.81	6	3.14	1	0.53	9	4.81
25:75	17	8.95	16	8.33	24	12.5	7	3.66	15	7.89	24	12.83
33:67	10	5.26	5	2.6	8	4.17	12	6.28	9	4.74	8	4.28
40:60	7	3.68	8	4.17	18	9.38	6	3.14	15	7.89	8	4.28
50:50	23	12.11	17	8.85	11	5.73	26	13.61	23	12.11	20	10.7
60:40	16	8.42	3	1.56	15	7.81	11	5.76	19	10.00	4	2.14
67:33	12	6.32	6	3.13	15	7.81	16	8.38	9	4.74	10	5.35
75:25	19	10.00	2	1.04	12	6.25	15	7.85	12	6.32	5	2.67
80:20	8	4.21	3	1.56	17	8.85	7	3.66	8	4.21	4	2.14
100:0	15	7.89	7	3.65	17	8.85	55	28.8	45	23.68	40	21.39

*HO* high oleic form of oilseed rape, *LL* low linolenic form of oilseed rape, *P* pathotypes of *Plasmodiophora brassicae* designated according to Somé et al. (1996), *Triplets* pathotypes of *P. brassicae* designated according to Buczacki et al. (1975)

**Fig. 7:**
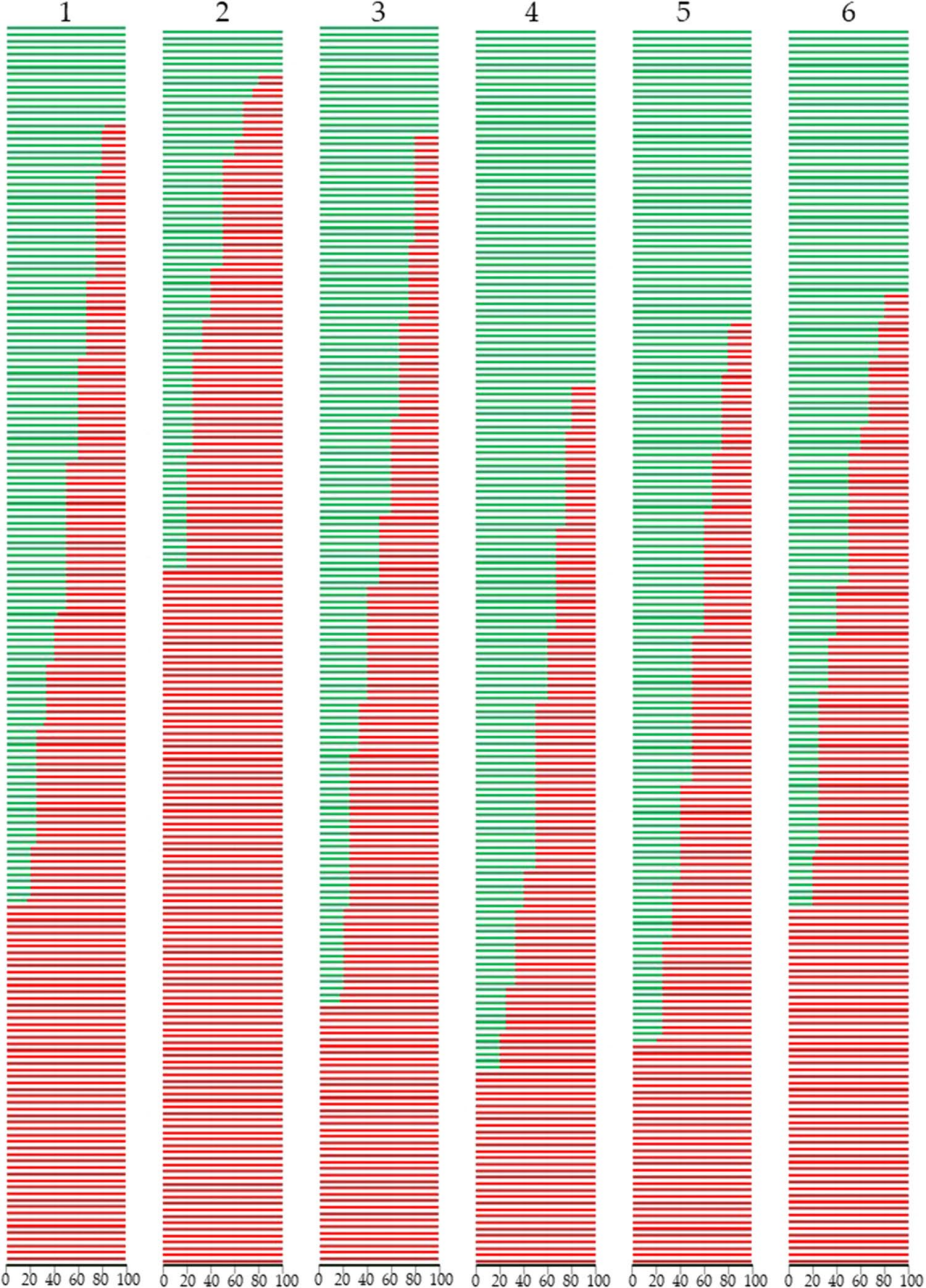
Resistance of the winter oilseed rape (*Brassica napus* L.) lines in the F3 progeny of the crosses between maternal lines with HO (high oleic) or LL (low linolenic) components and cv. Tosca to six isolates of *Plasmodiophora brassicae* (clubroot) listed in Table 4; line fully resistant (R, green bar), line fully susceptible (S, red bar), lines with plants segregating to resistant and susceptible are presented as green and red, the proportion of colors represents the proportion of R:S

The average percentage of fully susceptible lines was 28.1%, with 5.0% and 9.0% of lines having 80% and 75% of susceptible plants, respectively. Conversely, fewer lines were fully resistant (15.7%) with 4.1% and 5.7% of lines with the majority of resistant plants (Fig. 6). Among the tested progeny of 192 F_3_ generation lines, 10.5% displayed a 50:50 segregation of resistant to susceptible plants. The proportion between R and S plant response was pathotype-specific. The highest number of fully resistant lines was observed when testing with pathotype 4, while the highest number of fully susceptible lines was found when pathotype 2 was used to discriminate between resistance and susceptibility to clubroot (Table 3, Fig. 7).

Of the 192 lines tested, 57 (29.7%) were fully resistant to one pathotype, and 30 (15.6%) were fully resistant to two pathotypes. Thereafter, the number of lines resistant to more than two pathotypes rapidly decreased, with only one line resistant to five pathotypes (except for pathotype 5, 16/03/08), and no lines were resistant to all six isolates. The highest number of lines (14–18) was resistant to pathotypes 4 (P3, 16/14/12), 5 (P1, 16/03/08), and 6 (P2, 16/15/15). The same trend was observed for resistance to two or three pathotypes, as the three aforementioned pathotypes 4, 5, and 6 were also the most common in this group (Table 4).

**Table 4:** Number of lines of oilseed rape (*Brassica napus*) obtained from crosses between maternal lines with HO (high oleic) or LL (low linolenic) components and the canola-type clubroot-resistant cv. Tosca (*n* = 192) fully resistant (R100) to *Plasmodiophora brassicae* (clubroot)

Number of R100 lines resistant to the given number of pathotypes	Isolate/pathotype					
	1	2	3	4	5	6
	P3 16/02/29	P1 16/19/08	P3 16/02/29	P3 16/14/12	P1 16/03/08	P2 16/15/15
One	3	3	5	18	14	14
Two	7	2	3	19	16	13
Three	1	0	6	13	13	9
Four	3	1	3	3	3	3
Five	1	1	1	1	0	1

*n* = 192, testing was done using 192 lines of oilseed rape

*R100* lines fully resistant to *Plasmodiophora brassicae* (clubroot), *P* pathotypes of *P. brassicae* designated according to Somé et al. (1996), *Triplets* pathotypes of *P. brassicae* designated according to Buczacki et al. (1975)

### Altered fatty acids combined with clubroot resistance

The variants combining high oleic acid content (over 72%) and full resistance to at least one pathotype or high oleic acid content and low linolenic acid content (below 5%) along with full resistance to at least one pathotype comprised 9% of the entire population (18 lines out of 192), including 10 HO and 8 HOLL lines. In the HO group comprising 10 lines, 2 lines were resistant to a single pathotype, 2 lines were fully resistant to four pathotypes, and 3 lines were resistant to two or three pathotypes. None of the lines were fully resistant to all pathotypes. As regards the resistance to four pathotypes, the lines varied, with one harboring resistance to pathotypes 1 and 4–6 and another being fully resistant to pathotypes 3–6. Half of the HOLL lines (4) were fully resistant to only one pathotype, but there were also 2 lines resistant to three pathotypes and 3 lines resistant to four pathotypes (Table 5). Again, the lines resistant to four pathotypes differed: one line was fully resistant to pathotypes 4–6, whereas the vast majority of plants (80%) was resistant to pathotype 3; another line was fully resistant to pathotypes 1, 4, and 5, whereas most of the plants (80%) were resistant to pathotype 6. Different variants (combinations of resistance) were found in the remaining HO or HOLL lines showing resistance to various combinations of one, two, or three pathotypes. In spite of the designation to the same pathotype (P3, 16/02/29), the response of the material to isolates 1 and 3 was not identical which partially results from the segregation of the resistance trait but also suggests that these two systems of pathotype identification do not cover the whole genetic differentiation of *P. brassicae* isolates.

**Table 5:** Characterization of new HO-CR and HOLL-CR forms of winter oilseed rape (*Brassica napus*) with altered fatty acid composition and resistance to selected pathotypes of *Plasmodiophora brassicae* (clubroot)

No	Line no	FA content		Percentage of R/S plants in clubroot test												FA type	No. of *Pb* isolates
		Oleic C18:1	Linolenic C18:3	Isolate 1		Isolate 2		Isolate 3		Isolate 4		Isolate 5		Isolate 6			
				Pathotype P3 16/02/29		Pathotype P1 16/19/08		Pathotype P3 16/02/29		Pathotype P3 16/14/12		Pathotype P1 16/03/08		Pathotype P2 16/15/15			
				R	S	R	S	R	S	R	S	R	S	R	S		
1	48	76.9	7.1	50	50	0	100	40	60	50	50	40	60	100	0	HO	1
2	59	75.6	7.4	0	100	0	100	100	0	100	0	100	0	60	40	HO	3
3	62	76.3	6.5	60	40	0	100	100	0	100	0	100	0	100	0	HO	4
4	68	76.4	7.7	50	50	25	75	100	0	60	40	25	75	100	0	HO	2
5	76	75.5	8.1	50	50	50	50	80	20	100	0	75	25	25	75	HO	2
6	84	75.4	6.8	60	40	20	80	67	33	75	25	80	20	0	100	HO	1
7	97	72.7	8.8	50	50	50	50	60	40	100	0	100	0	100	0	HO	3
8	98	76.5	8.0	0	100	100	0	0	100	75	25	83	17	100	0	HO	3
9	110	78.3	7.7	0	100	80	20	20	80	0	100	100	0	0	100	HO	2
10	125	73.2	5.8	100	0	50	50	60	40	100	0	100	0	100	0	HO	4
11	150	72.4	4.6	25	75	0	100	80	20	100	0	100	0	100	0	HOLL	4
12	151	74.0	3.4	100	0	50	50	67	33	100	0	100	0	80	20	HOLL	4
13	152	78.8	4.2	67	33	0	100	67	33	100	0	50	50	50	50	HOLL	1
14	158	77.9	2.8	0	100	0	100	25	75	100	0	33	67	67	33	HOLL	1
15	161	73.9	2.9	67	33	0	100	33	67	100	0	100	0	100	0	HOLL	3
16	167	73.5	3.1	20	80	0	100	67	33	100	0	100	0	100	0	HOLL	3
17	168	72.3	4.3	0	100	100	0	0	100	0	100	60	40	50	50	HOLL	1
18	184	75.0	4.1	25	75	0	100	67	33	67	33	100	0	0	100	HOLL	1

*R* resistant, *S* susceptible, *HO-CR* oilseed rape line with high amount of oleic acid (≥ 72%) in seed oil and resistant to clubroot (CR)), *HOLL-CR* oilseed rape line with high content of oleic acid (≥ 72%) and low content of linolenic acid (< 5%) in seed oil and resistant to clubroot (CR), *No. of Pb isolates* number of isolates to which the clubroot resistance (CR) was demonstrated

## Discussion

In our study, we obtained highly valuable oilseed rape plants with a unique combination of fatty acids and resistance to selected *P. brassicae* pathotypes prevalent in Europe. Of the population of 80 lines with a high oleic acid content, 10 lines (12.5%) were resistant to at least one *P. brassicae* pathotype, with 2 lines (2.5%) resistant to four pathotypes. In addition, there were 30 lines with low linolenic acid content, 13 of which contained a high amount of oleic acid in seed oil. When the resistance to clubroot was added, the number of HOLL lines showing resistance narrowed down from 13 to 8, with 2 lines resistant to four pathotypes. Notably, two HO lines and two HOLL lines displayed resistance to the same set of pathotypes. To our knowledge, the oilseed rape plants obtained in our study represent a novel achievement, hitherto unreported in the existing literature.

One of the greatest challenges in research and breeding lies in the integration of altered fatty acid composition in seeds with disease resistance. Plant pests pose a substantial threat to agriculture, and advancements in genetic modifications and genome editing are expanding the breeders’ toolkit, providing means to control plant pathogens and insects (van Esse et al. 2020; Obermeier et al. 2022). While these methods are still under extensive discussion and not yet implemented in Europe, the introduction of genetic resistance to diseases from resistance sources through conventional means is considered highly desirable for sustainable agricultural crop production (Garrett et al. 2017). Resistance genes are a major highly promoted tool in integrated disease control. It is well recognized that pathogens can eventually overcome disease resistance and the effectiveness of resistance genes, and their duration vary, with some lasting only a few seasons, while others remaining effective for decades (McDonald and Linde 2002). A successful approach requires knowledge of the current pathogen population with an indication of projected future changes in the pathogen population. In this study, the modified composition of fatty acids was combined with resistance to at least one and up to four *P. brassicae* pathotypes infecting oilseed rape crops in Poland. Among the 192 recombinant lines, 106 (55.2%) exhibited resistance to at least one pathotype, while 49 (25.5%) demonstrated resistance to two or more of the five pathotypes tested in various combinations. This is consistent with numerous previous findings showing that clubroot resistance is pathotype-specific (Dixon 2014, Peng et al. 2014, Rahman et al. 2014, Niemann et al. 2017, Czajka et al. 2020, Dakouri et al. 2021). Notably, in our study, only one line was resistant to five isolates, whereas no line was resistant to all six isolates tested. The results of testing done in this study suggest that the systems of pathotype classification by Buczacki et al. (1975) and Somé et al. (1996) are insufficient to discriminate the genetic variation between the pathotypes of *P. brassicae*, even when the systems are used together.

This study demonstrated the feasibility of combining altered fatty acid contents and resistance to several *P. brassicae* pathotypes derived from the cv. Tosca. HO-CR and HOLL-CR lines combine the HO or HOLL traits with clubroot resistance (CR). Clubroot resistance in this cultivar originates from the QTL genomic region housing the *CRk*, *Crr3*, and *CRd* gene(s) (Fredua-Agyeman et al. 2021) with the local duplication of the *Crr3* gene (Kopeć et al. 2021). Based on the results of this study cv. Tosca was fully resistant to three isolates designated to two pathotypes, but its resistance to three other pathotypes from Europe was only partial or incomplete. This is why the progeny of these crossings resulted in fewer lines resistant to clubroot. This is in line with the findings of Fredua-Agyeman et al. (2021) who reported that clubroot resistance derived from the cv. Tosca was not effective against virulent *P. brassicae* isolates from Alberta, Canada. The differences in the pathogen population between Europe and Canada may hinder the use of these lines in fields infested with the Canadian population of the pathogen. Further studies are necessary to compare the isolates and identify differences between local populations. Although such studies have been conducted in Europe (Zamani-Noor et al 2021), no comparisons between Europe and Canada have been published to date. Characterizing *P. brassicae* populations would facilitate the selective exchange of cultivars with resistance to specific pathotypes of this damaging pathogen.

### Supplementary Information

Below is the link to the electronic supplementary material.

Supplementary file1 (DOCX 1.08 MB)

## Data Availability

The data that support the findings of this study are available from the first author and the corresponding author upon reasonable request.
