# Supplementary material for: HO-CR and HOLL-CR: new forms of winter oilseed rape (Brassica napus L.) with altered fatty acid composition and resistance to selected pathotypes of Plasmodiophora brassicae (clubroot)
Source: J Appl Genet. 2024 Apr 19;65(3):439–52. doi: 10.1007/s13353-024-00867-y (PMC11310246; doi:10.1007/s13353-024-00867-y)
Supplement: Supplementary file 1 — Supplementary file1 (DOCX 1.08 MB) [file 13353_2024_867_MOESM1_ESM.docx]

**HO-CR and HOLL-CR: new forms of winter oilseed rape (*Brassica napus* L.) with altered fatty acid composition and resistance to selected pathotypes of *Plasmodiophora brassicae* (clubroot)**

Stanisław Spasibionek^1^ (https://orcid.org/0000-0002-2853-312X), Katarzyna Mikołajczyk^1^ (https://orcid.org/0000-0002-5874-2213), Marcin Matuszczak^1^ (https://orcid.org/0000-0003-0712-7568), Joanna Kaczmarek^2^ (https://orcid.org/0000-0001-6437-5678), Noor Ramzi^2^ (<https://orcid.org/0000-0002-5466-6858>), Małgorzata Jędryczka^2#^ (https://orcid.org/0000-0001-8583-0772)

^1^Plant Breeding and Acclimatization Institute-National Research Institute, Poznań Division, Department of Oilseed Crops, Strzeszyńska 36, 60-479 Poznań, Poland

^2^Institute of Plant Genetics, Polish Academy of Sciences, Strzeszyńska 34, 60-479 Poznań, Poland

^#^  corresponding author: [mjed@igr.poznan.pl](mailto:mjed@igr.poznan.pl)

**SUPPLEMENTARY DATA**

Table S1. Characterisation of 29 forms of winter oilseed rape (*Brassica napus* L.) with altered fatty acid composition and resistance to the selected pathotypes of *Plasmodiophora brassicae* (clubroot) obtained in F_3_ generation after crossing the 2038 genotype (HO type) with the cultivar Tosca

| No. | FA content | | Percent of R/S plants in clubroot test | | | | | | | | | | | |
| --- | --- | --- | --- | --- | --- | --- | --- | --- | --- | --- | --- | --- | --- | --- |
|  | OleicC18:1 | LinolenicC18:3 | 1 | | 2 | | 3 | | 4 | | 5 | | 6 | |
|  |  |  | R | S | R | S | R | S | R | S | R | S | R | S |
| 1 | 66.8 | 10.1 | 75 | 25 | 0 | 100 | 80 | 20 | 0 | 100 | 100 | 0 | 67 | 33 |
| 2 | 69.4 | 9.0 | 25 | 75 | 25 | 75 | 67 | 33 | 50 | 50 | 100 | 0 | 25 | 75 |
| 3 | 70.3 | 8.9 | 40 | 60 | 50 | 50 | 80 | 20 | 33 | 67 | 33 | 67 | 25 | 75 |
| 4 | 68.8 | 9.0 | 25 | 75 | 0 | 100 | 0 | 100 | 25 | 75 | 75 | 25 | 100 | 0 |
| 5 | 70.7 | 8.8 | 50 | 50 | 0 | 100 | 25 | 75 | 33 | 67 | 33 | 67 | 67 | 33 |
| 6 | 70.0 | 9.8 | 33 | 67 | 0 | 100 | 80 | 20 | 75 | 25 | 60 | 40 | 67 | 33 |
| 7 | 70.6 | 8.5 | 0 | 100 | 50 | 50 | 60 | 40 | 67 | 33 | 100 | 0 | 25 | 75 |
| 8 | 70.0 | 8.9 | 50 | 50 | 20 | 80 | 33 | 67 | 40 | 60 | 25 | 75 | 100 | 0 |
| 9 | 66.7 | 9.8 | 67 | 33 | 50 | 50 | 60 | 40 | 50 | 50 | 40 | 60 | 50 | 50 |
| 10 | 67.1 | 10.1 | 50 | 50 | 0 | 100 | 60 | 40 | 33 | 67 | 0 | 100 | 75 | 25 |
| 11 | 68.0 | 9.2 | 25 | 75 | 40 | 60 | 40 | 60 | 80 | 20 | 25 | 75 | 100 | 0 |
| 12 | 72.6 | 8.6 | 60 | 40 | 20 | 80 | 40 | 60 | 80 | 20 | 100 | 0 | 0 | 100 |
| 13 | 70.3 | 9.0 | 75 | 25 | 40 | 60 | 75 | 25 | 80 | 20 | 0 | 100 | 100 | 0 |
| 14 | 68.3 | 9.0 | 50 | 50 | 0 | 100 | 25 | 75 | 40 | 60 | 25 | 75 | 0 | 100 |
| 15 | 67.6 | 8.7 | 67 | 33 | 0 | 100 | 20 | 80 | 67 | 33 | 40 | 60 | 60 | 40 |
| 16 | 69.2 | 8.8 | 60 | 40 | 0 | 100 | 20 | 80 | 50 | 50 | 33.3 | 67 | 0 | 100 |
| 17 | 72.4 | 8.4 | 31 | 69 | 20 | 80 | 33 | 66.7 | 100 | 0 | 60 | 40 | 80 | 20 |
| 18 | 68.8 | 9.3 | 60 | 40 | 25 | 75 | 40 | 60 | 33 | 67 | 0 | 100 | 100 | 0 |
| 19 | 72.9 | 8.1 | 50 | 50 | 0 | 100 | 60 | 40 | 20 | 80 | 25 | 75 | 0 | 100 |
| 20 | 68.8 | 7.3 | 25 | 75 | 0 | 100 | 40 | 60 | 50 | 50 | 0 | 100 | 25 | 75 |
| 21 | 69.6 | 7.2 | 75 | 25 | 0 | 100 | 100 | 0 | 67 | 33 | 50 | 50 | 50 | 50 |
| 22 | 71.8 | 8.1 | 0 | 100 | 0 | 100 | 0 | 100 | 0 | 100 | 0 | 100 | 40 | 60 |
| 23 | 71.4 | 8.0 | 0 | 100 | 0 | 100 | 0 | 100 | 0 | 100 | 0 | 100 | 20 | 80 |
| 24 | 76.9 | 7.1 | 50 | 50 | 0 | 100 | 40 | 60 | 50 | 50 | 40 | 60 | 100 | 0 |
| 25 | 73.7 | 6.8 | 75 | 25 | 0 | 100 | 67 | 33 | 100 | 0 | 75 | 25 | 100 | 0 |
| 26 | 74.0 | 7.4 | 25 | 75 | 20 | 80 | 80 | 20 | 80 | 20 | 33 | 67 | 40 | 60 |
| 27 | 71.5 | 7.9 | 0 | 100 | 0 | 100 | 0 | 100 | 0 | 100 | 0 | 100 | 0 | 100 |
| 28 | 72.5 | 7.4 | 80 | 20 | 0 | 100 | 80 | 20 | 80 | 20 | 67 | 33 | 50 | 50 |
| 29 | 65.9 | 7.4 | 25 | 75 | 0 | 100 | 40 | 60 | 40 | 60 | 0 | 100 | 100 | 0 |

# R ‒ Resistant, S – Susceptible, * ‒ no data

Table S2. Characterisation of 41 forms of winter oilseed rape (*Brassica napus* L.) with altered fatty acid composition and resistance to selected pathotypes of *Plasmodiophora brassicae* (clubroot) obtained in F_3_ generation after crossing the 2050 genotype (HO type) with the cultivar Tosca

| No. | FA content | | Percent of R/S plants in clubroot test | | | | | | | | | | | |
| --- | --- | --- | --- | --- | --- | --- | --- | --- | --- | --- | --- | --- | --- | --- |
|  | OleicC18:1 | LinolenicC18:3 | 1 | | 2 | | 3 | | 4 | | 5 | | 6 | |
|  |  |  | R | S | R | S | R | S | R | S | R | S | R | S |
| 1 | 69.0 | 8.8 | 43 | 57 | 20 | 80 | 33 | 67 | 60 | 40 | 80 | 20 | 25 | 75 |
| 2 | 71.2 | 8.9 | 33 | 67 | 0 | 100 | 20 | 80 | 60 | 40 | 100 | 0 | 25 | 75 |
| 3 | 72.0 | 8.8 | 20 | 80 | 0 | 100 | 20 | 80 | 0 | 100 | 40 | 60 | 25 | 75 |
| 4 | 78.1 | 6.3 | 0 | 100 | 40 | 60 | 17 | 83 | 20 | 80 | 25 | 75 | 60 | 40 |
| 5 | 69.7 | 10.2 | 50 | 50 | 0 | 100 | 100 | 0 | 67 | 33.3 | 60 | 40 | 0 | 100 |
| 6 | 77.2 | 6.6 | 50 | 50 | 25 | 75 | 50 | 50 | 75 | 25 | 25 | 75 | 20 | 80 |
| 7 | 66.3 | 7.0 | 0 | 100 | 25 | 75 | 40 | 60 | 75 | 25 | 100 | 0 | 100 | 0 |
| 8 | 73.7 | 6.3 | 0 | 100 | 25 | 75 | 25 | 75 | 33 | 67 | 25 | 75 | 25 | 75 |
| 9 | 75.6 | 7.4 | 0 | 100 | 0 | 100 | 100 | 0 | 100 | 0 | 100 | 0 | 60 | 40 |
| 10 | 69.7 | 8.6 | 40 | 60 | 0 | 100 | 50 | 50 | 100 | 0 | 40 | 60 | 33 | 67 |
| 11 | 74.1 | 8.4 | 0 | 100 | 0 | 100 | 25 | 75 | 33 | 67 | 75 | 25 | 20 | 80 |
| 12 | 76.3 | 6.5 | 60 | 40 | 0 | 100 | 100 | 0 | 100 | 0 | 100 | 0 | 100 | 0 |
| 13 | 72.1 | 4.6 | * | * | 0 | 100 | 0 | 100 | 67 | 33 | 75 | 25 | 67 | 33 |
| 14 | 78.0 | 7.5 | 0 | 100 | 50 | 50 | 50 | 50 | 75 | 25 | 60 | 40 | 20 | 80 |
| 15 | 77.3 | 7.1 | 0 | 100 | 20 | 80 | 0 | 100 | 0 | 100 | 0 | 100 | 0 | 100 |
| 16 | 77.7 | 6.4 | 83 | 17 | 25 | 75 | 60 | 40 | 75 | 25 | 60 | 40 | 50 | 50 |
| 17 | 74.3 | 7.5 | 0 | 100 | 0 | 100 | 40 | 60 | 0 | 100 | 0 | 100 | 0 | 100 |
| 18 | 76.4 | 7.7 | 50 | 50 | 25 | 75 | 100 | 0 | 60 | 40 | 25 | 75 | 100 | 0 |
| 19 | 67.1 | 8.5 | 0 | 100 | 0 | 100 | 25 | 75 | 20 | 80 | 0 | 100 | 0 | 100 |
| 20 | 63.7 | 7.9 | 60 | 40 | 0 | 100 | 50 | 50 | 100 | 0 | 80 | 20 | 33 | 67 |
| 21 | 69.5 | 8.7 | 33 | 67 | 0 | 100 | 17 | 83 | 20 | 80 | 0 | 100 | 0 | 100 |
| 22 | 67.4 | 7.5 | 100 | 0 | 100 | 0 | 100 | 0 | 50 | 50 | * | * | 100 | 0 |
| 23 | 70.6 | 9.5 | 100 | 0 | 33 | 67 | 60 | 40 | 50 | 50 | 60 | 40 | 33 | 67 |
| 24 | 78.9 | 6.3 | 0 | 100 | 0 | 100 | 75 | 25 | 50 | 50 | 67 | 33 | 20 | 80 |
| 25 | 69.1 | 10.0 | 60 | 40 | 0 | 100 | 80 | 20 | 100 | 0 | 100 | 0 | 50 | 50 |
| 26 | 75.5 | 8.1 | 50 | 50 | 50 | 50 | 80 | 20 | 100 | 0 | 75 | 25 | 25 | 75 |
| 27 | 79.8 | 6.8 | 0 | 100 | 20 | 80 | 40 | 60 | 20 | 80 | 0 | 100 | 0 | 100 |
| 28 | 78.2 | 7.7 | 0 | 100 | 0 | 100 | 0 | 100 | 25 | 75 | 0 | 100 | 0 | 100 |
| 29 | 64.7 | 9.4 | 75 | 25 | 20 | 80 | 25 | 75 | 40 | 60 | 80 | 20 | 25 | 75 |
| 30 | 71.9 | 7.9 | 60 | 40 | 0 | 100 | 40 | 60 | 100 | 0 | 80 | 20 | 22 | 78 |
| 31 | 73.0 | 7.9 | 80 | 20 | 0 | 100 | 25 | 75 | 100 | 0 | 100 | 0 | 25 | 75 |
| 32 | 74.6 | 7.4 | 80 | 20 | 20 | 80 | 75 | 25 | 75 | 25 | 50 | 50 | 33 | 67 |
| 33 | 73.4 | 6.6 | 75 | 25 | 0 | 100 | 75 | 25 | 100 | 0 | 100 | 0 | 0 | 100 |
| 34 | 75.4 | 6.8 | 60 | 40 | 20 | 80 | 67 | 33 | 75 | 25 | 80 | 20 | 0 | 100 |
| 35 | 67.8 | 8.8 | 0 | 100 | 0 | 100 | 20 | 80 | 0 | 100 | 0 | 100 | 25 | 75 |
| 36 | 78.7 | 6.7 | 25 | 75 | 0 | 100 | 0 | 100 | 33 | 67 | 0 | 100 | 0 | 100 |
| 37 | 72.8 | 8.2 | 20 | 80 | 0 | 100 | 0 | 100 | 25 | 75 | 0 | 100 | 25 | 75 |
| 38 | 75.9 | 7.2 | 80 | 20 | 0 | 100 | 25 | 75 | 60 | 40 | 40 | 60 | 25 | 75 |
| 39 | 65.1 | 9.6 | 0 | 100 | 0 | 100 | 0 | 100 | 0 | 100 | 0 | 100 | 0 | 100 |
| 40 | 77.3 | 8.5 | 0 | 100 | 25 | 75 | 0 | 100 | 0 | 100 | 0 | 100 | 0 | 100 |
| 41 | 71.2 | 8.5 | 0 | 100 | 0 | 100 | 0 | 100 | 75 | 25 | 0 | 100 | 0 | 100 |

# R ‒ Resistant, S – Susceptible, * ‒ no data

Table S3. Characterisation of 45 forms of winter oilseed rape (*Brassica napus* L.) with altered fatty acid composition and resistance to selected pathotypes of *Plasmodiophora brassicae* (clubroot) obtained in F_3_ generation after crossing the 2065 genotype (HO-type) with the cultivar Tosca

| No. | FA content | | Percent of R/S plants in clubroot test | | | | | | | | | | | |
| --- | --- | --- | --- | --- | --- | --- | --- | --- | --- | --- | --- | --- | --- | --- |
|  | OleicC18:1 | LinolenicC18:3 | 1 | | 2 | | 3 | | 4 | | 5 | | 6 | |
|  |  |  | R | S | R | S | R | S | R | S | R | S | R | S |
| 1 | 69.8 | 8.9 | 40 | 60 | 0 | 100 | 25 | 75 | 50 | 50 | 60 | 40 | 50 | 50 |
| 2 | 68.3 | 9.8 | 75 | 25 | 60 | 40 | 80 | 20 | 50 | 50 | 33 | 67 | 100 | 0 |
| 3 | 71.3 | 8.6 | 50 | 50 | 60 | 40 | 75 | 25 | 33 | 67 | 75 | 25 | 67 | 33 |
| 4 | 71.7 | 8.5 | 67 | 33 | 25 | 75 | 75 | 25 | 50 | 50 | 67 | 33 | 25 | 75 |
| 5 | 70.3 | 7.9 | * | * | 80 | 20 | 80 | 20 | 40 | 60 | 80 | 20 | 50 | 50 |
| 6 | 71.2 | 8.0 | 25 | 75 | 0 | 100 | 60 | 40 | 50 | 50 | 0 | 100 | 0 | 100 |
| 7 | 70.0 | 8.5 | 0 | 100 | 0 | 100 | 80 | 20 | 50 | 50 | 60 | 40 | 25 | 75 |
| 8 | 72.7 | 7.6 | 50 | 50 | 40 | 60 | 40 | 60 | 33 | 67 | 40 | 60 | 100 | 0 |
| 9 | 72.4 | 7.7 | 67 | 33 | 0 | 100 | 80 | 20 | 50 | 50 | 75 | 25 | 0 | 100 |
| 10 | 73.0 | 7.8 | 75 | 25 | 0 | 100 | 67 | 33 | 100 | 0 | 60 | 40 | 0 | 100 |
| 11 | 66.2 | 8.4 | 40 | 60 | 20 | 80 | 100 | 0 | 67 | 33 | 40 | 60 | 100 | 0 |
| 12 | 68.3 | 10.4 | 75 | 25 | 0 | 100 | 67 | 33 | 100 | 0 | 25 | 75 | 40 | 60 |
| 13 | 72.9 | 10.3 | 100 | 0 | 0 | 100 | 25 | 75 | 100 | 0 | 50 | 50 | 0 | 100 |
| 14 | 72.7 | 8.8 | 50 | 50 | 50 | 50 | 60 | 40 | 100 | 0 | 100 | 0 | 100 | 0 |
| 15 | 76.5 | 8.0 | 0 | 100 | 100 | 0 | 0 | 100 | 75 | 25 | 83 | 17 | 100 | 0 |
| 16 | 66.9 | 7.4 | 50 | 50 | 0 | 100 | 80 | 20 | 100 | 0 | 100 | 0 | 25 | 75 |
| 17 | 77.1 | 8.4 | 0 | 100 | 0 | 100 | 0 | 100 | 0 | 100 | 0 | 100 | 0 | 100 |
| 18 | 62.2 | 9.0 | 25 | 75 | 50 | 50 | 25 | 75 | 75 | 25 | 50 | 50 | 40 | 60 |
| 19 | 71.5 | 8.7 | 100 | 0 | 67 | 33 | 67 | 33.3 | 60 | 40 | 100 | 0 | * | * |
| 20 | 65.0 | 8.3 | 33 | 67 | 25 | 75 | 60 | 40 | 60 | 40 | 50 | 50 | 100 | 0 |
| 21 | 66.6 | 7.6 | 0 | 100 | 100 | 0 | 0 | 100 | 0 | 100 | 40 | 60 | 50 | 50 |
| 22 | 79.4 | 7.1 | 75 | 25 | 50 | 50 | 20 | 80 | 60 | 40 | 25 | 75 | 60 | 40 |
| 23 | 66.9 | 7.9 | 0 | 100 | 50 | 50 | 20 | 80 | 33 | 67 | 40 | 60 | 20 | 80 |
| 24 | 72.7 | 7.9 | 0 | 100 | 0 | 100 | 40 | 60 | 50 | 50 | 100 | 0 | 100 | 0 |
| 25 | 71.6 | 7.0 | 75 | 25 | 67 | 33 | 0 | 100 | 100 | 0 | 50 | 50 | 100 | 0 |
| 26 | 70.8 | 9.0 | 25 | 75 | 0 | 100 | 25 | 75 | 60 | 40 | 100 | 0 | 50 | 50 |
| 27 | 78.3 | 7.7 | 0 | 100 | 80 | 20 | 20 | 80 | 0 | 100 | 100 | 0 | 0 | 100 |
| 28 | 74.8 | 6.9 | 50 | 50 | 50 | 50 | 40 | 60 | 80 | 20 | 100 | 0 | 100 | 0 |
| 29 | 69.1 | 6.9 | 0 | 100 | 75 | 25 | 0 | 100 | 100 | 0 | 100 | 0 | 0 | 100 |
| 30 | 67.8 | 8.1 | 0 | 100 | 0 | 100 | 0 | 100 | 0 | 100 | 40 | 60 | 80 | 20 |
| 31 | 72.5 | 8.2 | 25 | 75 | 0 | 100 | 75 | 25 | 100 | 0 | 80 | 20 | 100 | 0 |
| 32 | 73.0 | 7.8 | 50 | 50 | 50 | 50 | 25 | 75 | 67 | 33 | 100 | 0 | 100 | 0 |
| 33 | 69.4 | 8.4 | 60 | 40 | 33 | 67 | 100 | 0 | 75 | 25 | 50 | 50 | 50 | 50 |
| 34 | 70.3 | 8.5 | 0 | 100 | 0 | 100 | 25 | 75 | 0 | 100 | 50 | 50 | 100 | 0 |
| 35 | 69.7 | 6.2 | 0 | 100 | 25 | 75 | 33 | 67 | 100 | 0 | 50 | 50 | 0 | 100 |
| 36 | 70.1 | 6.4 | 0 | 100 | 0 | 100 | 0 | 100 | 0 | 100 | 33 | 67 | 0 | 100 |
| 37 | 74.2 | 7.0 | 75 | 25 | 0 | 100 | 60 | 40 | 100 | 0 | 75 | 25 | * | * |
| 38 | 74.8 | 6.0 | 75 | 25 | 0 | 100 | 25 | 75 | 67 | 33 | 60 | 40 | 50 | 50 |
| 39 | 73.1 | 8.2 | 80 | 20 | 60 | 40 | 67 | 33 | 50 | 50 | 67 | 33 | 0 | 100 |
| 40 | 82.6 | 5.4 | 0 | 100 | 0 | 100 | 0 | 100 | 0 | 100 | 0 | 100 | 0 | 100 |
| 41 | 75.6 | 6.0 | 40 | 60 | 20 | 80 | 25 | 75 | 50 | 50 | 25 | 75 | 0 | 100 |
| 42 | 73.2 | 5.8 | 100 | 0 | 50 | 50 | 60 | 40 | 100 | 0 | 100 | 0 | 100 | 0 |
| 43 | 72.1 | 5.5 | 25 | 75 | 25 | 75 | 25 | 75 | 0 | 100 | 60 | 40 | 100 | 0 |
| 44 | 65.5 | 7.7 | 100 | 0 | 0 | 100 | 33 | 67 | 67 | 33 | 67 | 33 | 33 | 67 |
| 45 | 70.7 | 7.4 | 20 | 80 | 100 | 0 | 0 | 100 | 25 | 75 | 0 | 100 | 0 | 100 |

# R ‒ Resistant, S – Susceptible, * ‒ no data

Table S4. Characterisation of 77 forms of winter oilseed rape (*Brassica napus* L.) with altered fatty acid composition and resistance to selected pathotypes of *Plasmodiophora brassicae* (clubroot) obtained in F_3_ generation after crossing the 2103 genotype (HOLL-type) with the cultivar Tosca

| No. | FA content | | Percent of R/S plants in clubroot test | | | | | | | | | | | |
| --- | --- | --- | --- | --- | --- | --- | --- | --- | --- | --- | --- | --- | --- | --- |
|  | OleicC18:1 | LinolenicC18:3 | 1 | | 2 | | 3 | | 4 | | 5 | | 6 | |
|  |  |  | R | S | R | S | R | S | R | S | R | S | R | S |
| 1 | 70.1 | 7.6 | 80 | 20 | 40 | 60 | 75 | 25 | 100 | 0 | 100 | 0 | 50 | 50 |
| 2 | 64.4 | 6.2 | 40 | 60 | 40 | 60 | 33 | 67 | 33 | 67 | 50 | 50 | 75 | 25 |
| 3 | 70.1 | 6.0 | 25 | 75 | 50 | 50 | 80 | 20 | 0 | 100 | 25 | 75 | 0 | 100 |
| 4 | 72.2 | 5.1 | 33 | 67 | 0 | 100 | 0 | 100 | 0 | 100 | 50 | 50 | 25 | 75 |
| 5 | 71.4 | 5.6 | 80 | 20 | 0 | 100 | 100 | 0 | 50 | 50 | 50 | 50 | 0 | 100 |
| 6 | 70.4 | 5.7 | 33 | 67 | 40 | 60 | 40 | 60 | 50 | 50 | 100 | 0 | * | * |
| 7 | 70.3 | 5.6 | 100 | 0 | 25 | 75 | 40 | 60 | 75 | 25 | 33 | 67 | 100 | 0 |
| 8 | 69.1 | 6.1 | 100 | 0 | 0 | 100 | 50 | 50 | 100 | 0 | 0 | 100 | 50 | 50 |
| 9 | 72.1 | 5.6 | 25 | 75 | 0 | 100 | 25 | 75 | 100 | 0 | 40 | 60 | 25 | 75 |
| 10 | 72.0 | 5.9 | 67 | 33 | 0 | 100 | 20 | 80 | 50 | 50 | 100 | 0 | 0 | 100 |
| 11 | 72.3 | 5.3 | 20 | 80 | 67 | 33 | 80 | 20 | 50 | 50 | 67 | 33 | 100 | 0 |
| 12 | 69.5 | 5.5 | 75 | 25 | 40 | 60 | 75 | 25 | 100 | 0 | 0 | 100 | 100 | 0 |
| 13 | 70.0 | 5.4 | 50 | 50 | 0 | 100 | 67 | 33 | 67 | 33 | 50 | 50 | 40 | 60 |
| 14 | 69.8 | 5.6 | 17 | 83 | 0 | 100 | 0 | 100 | 0 | 100 | 0 | 100 | 0 | 100 |
| 15 | 74.7 | 6.1 | 20 | 80 | 0 | 100 | 75 | 25 | 67 | 33 | 50 | 50 | 100 | 0 |
| 16 | 68.2 | 7.2 | 60 | 40 | 20 | 80 | 50 | 50 | 60 | 40 | 100 | 0 | 67 | 33 |
| 17 | 74.3 | 5.9 | 0 | 100 | 50 | 50 | 20 | 80 | 100 | 0 | 60 | 40 | 100 | 0 |
| 18 | 69.1 | 7.0 | 0 | 100 | 0 | 100 | 0 | 100 | 0 | 100 | 0 | 100 | 0 | 100 |
| 19 | 67.1 | 6.9 | 50 | 50 | 0 | 100 | 0 | 100 | 0 | 100 | 0 | 100 | 0 | 100 |
| 20 | 80.2 | 5.6 | 100 | 0 | 20 | 80 | 100 | 0 | 100 | 0 | 100 | 0 | 0 | 100 |
| 21 | 65.3 | 4.8 | 100 | 0 | 25 | 75 | 40 | 60 | 100 | 0 | 60 | 40 | 0 | 100 |
| 22 | 70.9 | 4.3 | 60 | 40 | 0 | 100 | 50 | 50 | 100 | 0 | 60 | 40 | 0 | 100 |
| 23 | 66.5 | 5.0 | 100 | 0 | 67 | 33 | 20 | 80 | 67 | 33 | 60 | 40 | 50 | 50 |
| 24 | 66.8 | 3.6 | 0 | 100 | 0 | 100 | 0 | 100 | 20 | 80 | 50 | 50 | 0 | 100 |
| 25 | 65.9 | 6.8 | 67 | 33 | 20 | 80 | 60 | 40 | 100 | 0 | 100 | 0 | 100 | 0 |
| 26 | 65.9 | 3.8 | 60 | 40 | 0 | 100 | 25 | 75 | 100 | 0 | 60 | 40 | 0 | 100 |
| 27 | 63.9 | 4.0 | 75 | 25 | 50 | 50 | 0 | 100 | 50 | 50 | 0 | 100 | 50 | 50 |
| 28 | 66.9 | 5.3 | 67 | 33 | 33 | 67 | 100 | 0 | 100 | 0 | 100 | 0 | 25 | 75 |
| 29 | 66.1 | 5.5 | 60 | 40 | 0 | 100 | 0 | 100 | 67 | 33 | 25 | 75 | 75 | 25 |
| 30 | 67.4 | 5.6 | 67 | 33 | 0 | 100 | 50 | 50 | 0 | 100 | 100 | 0 | 50 | 50 |
| 31 | 65.6 | 5.2 | 40 | 60 | 75 | 25 | 75 | 25 | 100 | 0 | 100 | 0 | 100 | 0 |
| 32 | 62.2 | 3.7 | 50 | 50 | 0 | 100 | 50 | 50 | 100 | 0 | 100 | 0 | * | * |
| 33 | 70.1 | 5.3 | 0 | 100 | 20 | 80 | 25 | 75 | 80 | 20 | 100 | 0 | 75 | 25 |
| 34 | 72.0 | 4.5 | 0 | 100 | 25 | 75 | 60 | 40 | 60 | 40 | 40 | 60 | 0 | 100 |
| 35 | 72.4 | 4.6 | 25 | 75 | 0 | 100 | 80 | 20 | 100 | 0 | 100 | 0 | 100 | 0 |
| 36 | 74.0 | 3.4 | 100 | 0 | 50 | 50 | 67 | 33 | 100 | 0 | 100 | 0 | 80 | 20 |
| 37 | 78.8 | 4.2 | 67 | 33 | 0 | 100 | 67 | 33 | 100 | 0 | 50 | 50 | 50 | 50 |
| 38 | 77.6 | 5.3 | 0 | 100 | 0 | 100 | 0 | 100 | 0 | 100 | 0 | 100 | 0 | 100 |
| 39 | 71.8 | 3.0 | 0 | 100 | 25 | 75 | 0 | 100 | * | * | 75 | 25 | 100 | 0 |
| 40 | 69.4 | 5.5 | 60 | 40 | 0 | 100 | 50 | 50 | 67 | 33 | 40 | 60 | 50 | 50 |
| 41 | 75.7 | 4.0 | 0 | 100 | 0 | 100 | 67 | 33 | 50 | 50 | 0 | 100 | 67 | 33 |
| 42 | 71.6 | 5.7 | 0 | 100 | 0 | 100 | 40 | 60 | 75 | 25 | 50 | 50 | 50 | 50 |
| 43 | 77.9 | 2.8 | 0 | 100 | 0 | 100 | 25 | 75 | 100 | 0 | 33 | 67 | 67 | 33 |
| 44 | 77.4 | 5.7 | 0 | 100 | 20 | 80 | 100 | 0 | 75 | 25 | 50 | 50 | 40 | 60 |
| 45 | 79.5 | 5.2 | 20 | 80 | 20 | 80 | 100 | 0 | 50 | 50 | 100 | 0 | 0 | 100 |
| 46 | 73.9 | 2.9 | 67 | 33 | 0 | 100 | 33 | 67 | 100 | 0 | 100 | 0 | 100 | 0 |
| 47 | 69.5 | 5.0 | 0 | 100 | 0 | 100 | 0 | 100 | 0 | 100 | 25 | 75 | 75 | 25 |
| 48 | 73.9 | 5.0 | 25 | 75 | 0 | 100 | 100 | 0 | 100 | 0 | 100 | 0 | 75 | 25 |
| 49 | 65.8 | 5.2 | 100 | 0 | 100 | 0 | 100 | 0 | 100 | 0 | * | * | 100 | 0 |
| 50 | 72.2 | 3.8 | 25 | 75 | 0 | 100 | 50 | 50 | 60 | 40 | 50 | 50 | 25 | 75 |
| 51 | 70.8 | 8.8 | 0 | 100 | 0 | 100 | 25 | 75 | 0 | 100 | 0 | 100 | * | * |
| 52 | 73.5 | 3.1 | 20 | 80 | 0 | 100 | 67 | 33 | 100 | 0 | 100 | 0 | 100 | 0 |
| 53 | 72.3 | 4.3 | 0 | 100 | 100 | 0 | 0 | 100 | 0 | 100 | 60 | 40 | 50 | 50 |
| 54 | 73.5 | 5.9 | 33 | 67 | 0 | 100 | 80 | 20 | 100 | 0 | 67 | 33 | 100 | 0 |
| 55 | 70.4 | 4.4 | 33 | 67 | 0 | 100 | 67 | 33 | 100 | 0 | 33 | 67 | 67 | 33 |
| 56 | 72.0 | 4.9 | 0 | 100 | 0 | 100 | 20 | 80 | 0 | 100 | 50 | 50 | 0 | 100 |
| 57 | 71.9 | 4.1 | 0 | 100 | 33 | 67 | 0 | 100 | 25 | 75 | 67 | 33 | 0 | 100 |
| 58 | 75.8 | 4.5 | 0 | 100 | 0 | 100 | 75 | 25 | 75 | 25 | 60 | 40 | 25 | 75 |
| 59 | 70.1 | 6.3 | 75 | 25 | 33 | 67 | 100 | 0 | 100 | 0 | 100 | 0 | 33 | 67 |
| 60 | 78.4 | 4.3 | 50 | 50 | 67 | 33 | 0 | 100 | 25 | 75 | 40 | 60 | 40 | 60 |
| 61 | 72.0 | 6.1 | 67 | 33 | 0 | 100 | 0 | 100 | 50 | 50 | 0 | 100 | 0 | 100 |
| 62 | 77.7 | 6.2 | 60 | 40 | 0 | 100 | 0 | 100 | 25 | 75 | 50 | 50 | 80 | 20 |
| 63 | 68.4 | 3.8 | 50 | 50 | 0 | 100 | 40 | 60 | 100 | 0 | 100 | 0 | 0 | 100 |
| 64 | 64.7 | 6.0 | 100 | 0 | 100 | 0 | 60 | 40 | 50 | 50 | 75 | 25 | 0 | 100 |
| 65 | 73.3 | 5.0 | 50 | 50 | 0 | 100 | 20 | 80 | 100 | 0 | 75 | 25 | 20 | 80 |
| 66 | 70.7 | 5.1 | 0 | 100 | 0 | 100 | 0 | 100 | 67 | 33 | 60 | 40 | 25 | 75 |
| 67 | 66.2 | 4.0 | 0 | 100 | 0 | 100 | 25 | 75 | 40 | 60 | 25 | 75 | 0 | 100 |
| 68 | 64.2 | 4.3 | 60 | 40 | 50 | 50 | 25 | 75 | 100 | 0 | 75 | 25 | 40 | 60 |
| 69 | 75.0 | 4.1 | 25 | 75 | 0 | 100 | 67 | 33 | 67 | 33 | 100 | 0 | 0 | 100 |
| 70 | 70.9 | 4.7 | 20 | 80 | 0 | 100 | 67 | 33 | 100 | 0 | 100 | 0 | 0 | 100 |
| 71 | 76.5 | 5.8 | 0 | 100 | 0 | 100 | 0 | 100 | 100 | 0 | 50 | 50 | 33 | 67 |
| 72 | 69.5 | 5.7 | 50 | 50 | 0 | 100 | 33 | 67 | 100 | 0 | 100 | 0 | 100 | 0 |
| 73 | 70.1 | 8.0 | 0 | 100 | 0 | 100 | 0 | 100 | 33 | 67 | 67 | 33 | 33 | 67 |
| 74 | 66.2 | 4.5 | 0 | 100 | 0 | 100 | 0 | 100 | 0 | 100 | 20 | 80 | 0 | 100 |
| 75 | 73.9 | 6.0 | 33 | 67 | 67 | 33 | 60 | 40 | 100 | 0 | 100 | 0 | 25 | 75 |
| 76 | 63.2 | 4.6 | 100 | 0 | 0 | 100 | 80 | 20 | 100 | 0 | 50 | 50 | 20 | 80 |
| 77 | 73.0 | 5.9 | 67 | 33 | 0 | 100 | 100 | 0 | 100 | 0 | 100 | 0 | 67 | 33 |

# R ‒ Resistant, S – Susceptible, * ‒ no data

#
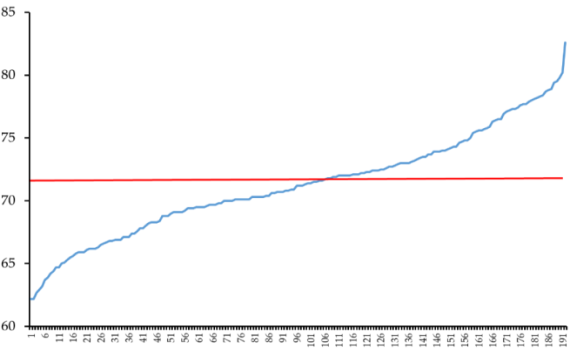

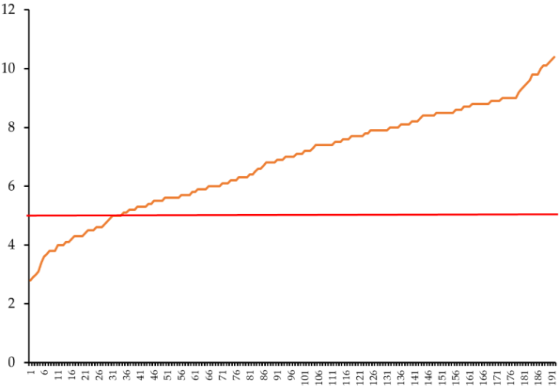


Low Linolenic LL lines

High Oleic HO lines

**B**

**A**

# Figure S1. Content of oleic acid, C18:1 (A) and linolenic acid, C18:3 (B) in the studied population of 192 recombinant lines of oilseed rape resulting from crosses between HO and HOLL lines of oilseed rape and cv. Tosca.

#
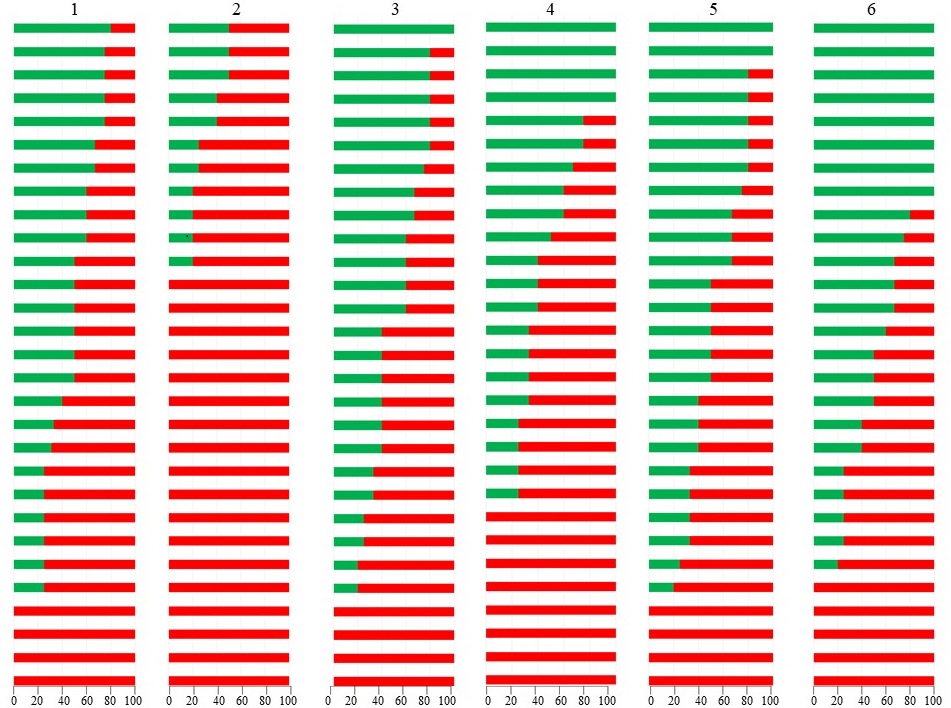


# Figure S2. Resistance of the winter oilseed rape (*Brassica napus* L.) lines in the F_3_ progeny of the genotype 2038: HOmut(M10464)×Contact crossed with cv. Tosca, to six isolates of *Plasmodiophora brassicae* (clubroot) listed in Table 4; line fully resistant (R, green bar), line fully susceptible (S, red bar), lines with plants segregating to resistant and susceptible are presented as green and red, the proportion of colors represents the proportion of R:S.

#
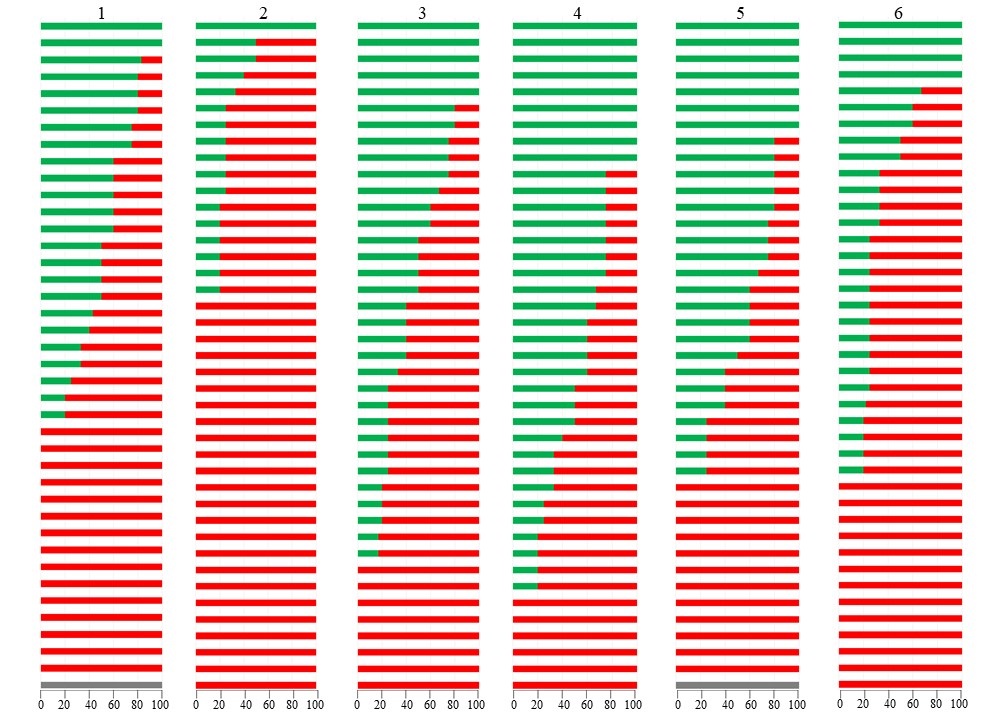


# Figure S3. Resistance of the winter oilseed rape (*Brassica napus* L.) lines in the F_3_ progeny of the genotype 2050: HOmut (M10453)×Contact crossed with cv. Tosca, to six isolates of *Plasmodiophora brassicae* (clubroot) listed in Table 4; line fully resistant (R, green bar), line fully susceptible (S, red bar), lines with plants segregating to resistant and susceptible are presented as green and red, the proportion of colors represents the proportion of R:S. Grey bar: no data for the line.

#

#
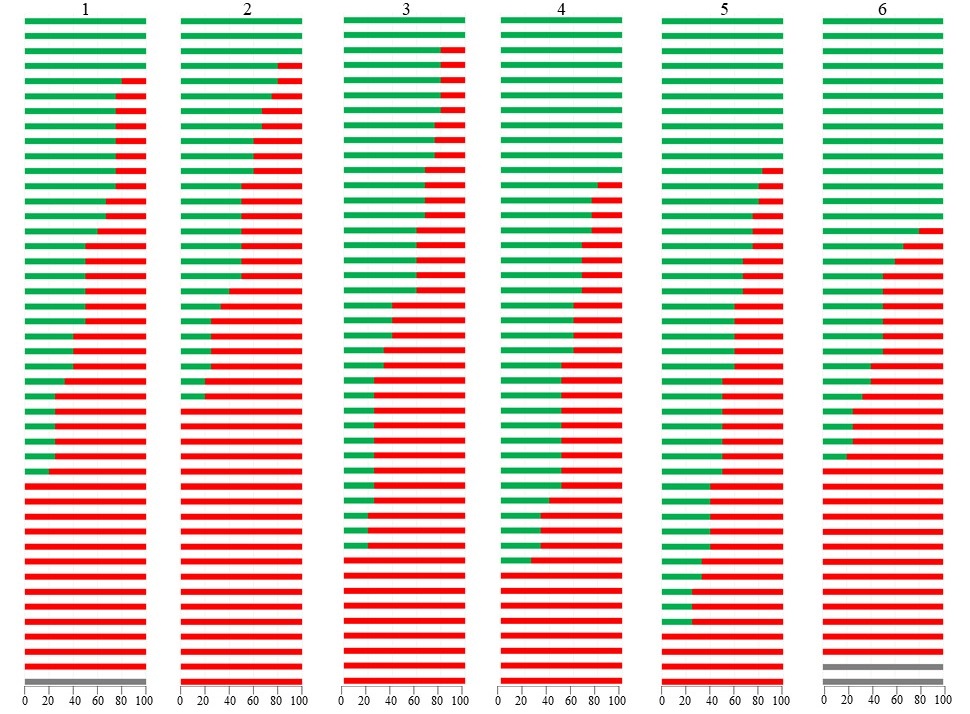


# Figure S4. Resistance of the winter oilseed rape (*Brassica napus* L.) lines in the F_3_ progeny of the genotype 2065: Californium×HOmut (M10464) crossed with cv. Tosca, to six isolates of *Plasmodiophora brassicae* (clubroot) listed in Table 4; line fully resistant (R, green bar), line fully susceptible (S, red bar), lines with plants segregating to resistant and susceptible are presented as green and red, the proportion of colors represents the proportion of R:S. Grey bar: no data for the line.

#

**
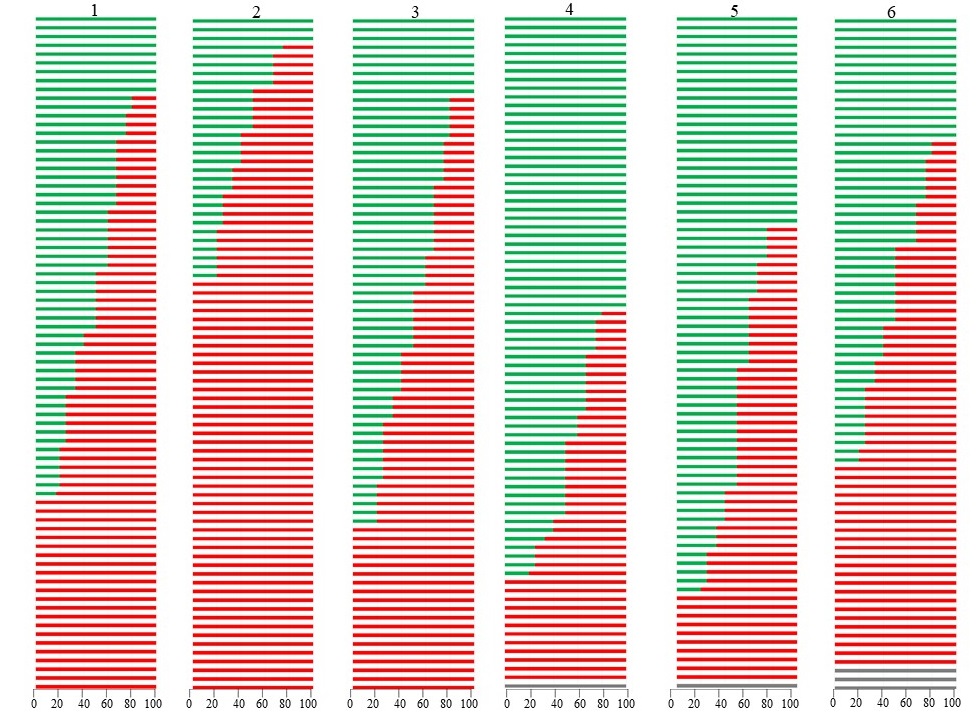
**

# Figure S5. Resistance of the winter oilseed rape (*Brassica napus* L.) lines in the F_3_ progeny of the genotype 2103: LLmut (M681)×HOmut (M10464) crossed with cv. Tosca, to six isolates of *Plasmodiophora brassicae* (clubroot) listed in Table 4; line fully resistant (R, green bar), line fully susceptible (S, red bar), lines with plants segregating to resistant and susceptible are presented as green and red, the proportion of colors represents the proportion of R:S. Grey bar: no data for the line.
